# A deep multi-task learning approach to identifying mummy berry infection sites, the disease stage, and severity

**DOI:** 10.3389/fpls.2024.1340884

**Published:** 2024-03-28

**Authors:** Hongchun Qu, Chaofang Zheng, Hao Ji, Rui Huang, Dianwen Wei, Seanna Annis, Francis Drummond

**Affiliations:** ^1^Institute of Ecological Safety and College of Automation, Chongqing University of Posts and Telecommunications, Chongqing, China; ^2^College of Computer Science, Chongqing University of Posts and Telecommunications, Chongqing, China; ^3^Institute of Natural Resources and Ecology, Heilongjiang Academy of Sciences, Harbin, China; ^4^School of Biology and Ecology, University of Maine, Orono, ME, United States; ^5^Cooperative Extension, University of Maine, Orono, ME, United States

**Keywords:** mummy berry disease, multi-task learning, transfer learning, convolutional neural network, blueberry, *Monilinia vaccinii-corymbosi*, *Vaccinium angustifolium*

## Abstract

**Introduction:**

Mummy berry is a serious disease that may result in up to 70 percent of yield loss for lowbush blueberries. Practical mummy berry disease detection, stage classification and severity estimation remain great challenges for computer vision-based approaches because images taken in lowbush blueberry fields are usually a mixture of different plant parts (leaves, bud, flowers and fruits) with a very complex background. Specifically, typical problems hindering this effort included data scarcity due to high manual labelling cost, tiny and low contrast disease features interfered and occluded by healthy plant parts, and over-complicated deep neural networks which made deployment of a predictive system difficult.

**Methods:**

Using real and raw blueberry field images, this research proposed a deep multi-task learning (MTL) approach to simultaneously accomplish three disease detection tasks: identification of infection sites, classification of disease stage, and severity estimation. By further incorporating novel superimposed attention mechanism modules and grouped convolutions to the deep neural network, enabled disease feature extraction from both channel and spatial perspectives, achieving better detection performance in open and complex environments, while having lower computational cost and faster convergence rate.

**Results:**

Experimental results demonstrated that our approach achieved higher detection efficiency compared with the state-of-the-art deep learning models in terms of detection accuracy, while having three main advantages: 1) field images mixed with various types of lowbush blueberry plant organs under a complex background can be used for disease detection; 2) parameter sharing among different tasks greatly reduced the size of training samples and saved 60% training time than when the three tasks (data preparation, model development and exploration) were trained separately; and 3) only one-sixth of the network parameter size (23.98M vs. 138.36M) and one-fifteenth of the computational cost (1.13G vs. 15.48G FLOPs) were used when compared with the most popular Convolutional Neural Network VGG16.

**Discussion:**

These features make our solution very promising for future mobile deployment such as a drone carried task unit for real-time field surveillance. As an automatic approach to fast disease diagnosis, it can be a useful technical tool to provide growers real time disease information that can prevent further disease transmission and more severe effects on yield due to fruit mummification.

## 1 Introduction

Lowbush blueberry or wild blueberry (*Vaccinium angustifolium* Aiton), is a North American native berry crop which is both economically and culturally important in Maine and other Northern New England states in the US, the Canadian Maritime provinces and the province of Quebec in Canada. The lowbush blueberry production system can be a major source of income for growers in this region (Hanes et al., 2015). The state of Maine is the world’s largest producer of wild blueberry (Obsie et al., 2020). Nearly 67.7 million pounds were produced in 2017 (*nass.usda.gov*). However, lowbush blueberry is highly susceptible to mummy berry disease caused by the fungus *Monilinia vaccinii-corymbosi* (Read) (MVC) (Penman and Annis, 2005). Mummy berry is a serious disease for many *Vaccinium* species and can cause up to 70% of yield loss, especially in the moist oceanic climate where it grows in Maine and Maritime Canada, posing an economic challenge to growers and affecting local economies (Hanes et al., 2015).

Correctly identifying mummy berry infection in its early stages and providing a reasonable estimation of disease severity could play a vital role in efficient disease control and prevention in the future. Because management procedures such as determining the timing and dose of fungicide sprays, as well as the introduction of commercially available honey bees (Qu and Drummond, 2018) relies upon sufficient information regarding where, when and how blueberry plants are infected (Penman and Annis, 2005). Specific information on the identification of infection sites on leaves, flowers or fruits; classification of infection stages; and estimation of disease severity will be useful in managing mummy berry disease. Considering the range in size of lowbush blueberry fields, which can be up to hundreds of hectares, automatic mummy berry infection identification techniques such as Computer Vision and Image Processing (CVIP) (Vishnoi et al., 2021) are advantageous, not only because of their extensive labor-saving potential, but also because of the potential comprehensive disease relevant measurements that can be used in directing chemical treatments across large crop land areas over time (Bock et al., 2010). In the near future, a drone carried disease detection task unit based on CVIP would be a very efficient tool for field disease surveillance (Liu and Wang, 2021).

Computer Vision and Image Processing methods have previously been used in a wide variety of diagnosis applications in precision agriculture such as plant species classification, leaf disease recognition, and plant disease severity estimation (Liang et al., 2019). In the last decade, many traditional machine learning models were proposed for the detection and classification of plant diseases. Rumpf et al. (2010) studied the early diagnosis and classification of diseases infecting sugar beet based on spectral plant indexes using Support Vector Machine (SVM). Ramesh et al. (2018) proposed a disease classification method using Random Forest algorithms to identify healthy and diseased images. Waghmare et al. (2016) proposed a Multiclass Support Vector Machine as a classification model for grape leaves and they identified diseases like Black rot with a reported accuracy of 96.6%.

Nevertheless, these traditional CVIP methods are heavily dependent on experience such as manual selection of disease spot features plus artificial classifiers, which inevitably lead to objective disease feature extraction (Sastry and Zitter, 2014). In the real environment, challenges, such as low contrast, high noise of lesions with respect to the background, large variations in size and scale of the target area, unstable illumination conditions, and image capture shooting angle, can compromise the practicality of these conventional CVIP methods (Alruily, 2021). In contrast, deep learning approaches, due to their excellent automatic feature engineering and self-learning capabilities, have resulted in state-of-the-art performance when compared to traditional CVIP approaches in different domains (Alom et al., 2019). Among deep learning methods, Convolutional Neural Networks (CNN) have shown extraordinary performance in image recognition tasks (Fuentes et al., 2017). Geetharamani and Arun Pandian (2019) trained a 9-layer CNN architecture on the PlantVillage dataset with different epoch, batch size and dropout rate. In the performance comparison with popular transfer learning approaches, the proposed model achieved 96.5% classification accuracy on the test dataset. Lu et al. (2017) proposed a technique to enhance the identification ability of CNNs to effectively classify 10 rice diseases through deep convolution neural networks. A similar approach was also developed by Ferentinos (2018) to recognize and diagnose plant diseases based on simple leaf images of healthy and diseased crops. In addition, Fuentes et al. (2017) developed a practical solution based on a robust CNNs-based detector for real-time recognition of diseases and pests of tomato plants.

Although the existing deep learning techniques have achieved significant success in plant disease detection and diagnosis, they usually train a single model or a set of separate models to solve a specific task. For example, when addressing the two tasks, such as plant disease identification and severity estimation, it is common to train two separate models to optimize their respective performance metrics. This idea of problem solving is called single-task learning (STL). Single-task learning ignores over-lapping information between related tasks that might be very helpful for enhancing model generality and promoting model performance. Alternatively, multi-task learning (MTL) can train a system capable of solving multiple tasks simultaneously by sharing representations between them (Ruder, 2017). By doing so, MTL can not only achieve higher detection accuracy than STL by learning joint generalized representations (Zhang and Yang, 2017), but can also sufficiently decrease overfitting risk by using the domain information contained in the training signals of related tasks as an inductive bias. Furthermore, the joint training of MTL can also shrink the size of training data (equivalent to data augmentation), which is critical in dealing with data deficient problems (Caruana, 1997).

To simultaneously identify mummy berry infection sites, classify disease stages and estimate the severity level for real farming conditions is difficult. Unlike controllable conditions in the laboratory, field-taken images of lowbush blueberry usually have mixed plant parts (leaves, buds, flowers and fruits) embedded in a very complex background with low contrast posing great challenges for current deep learning models. The first one is data scarcity due to the extremely high manual labelling cost. This is the major obstacle for training deep learning models for different tasks. The second is the tiny, low contrast disease features that interfered with and occluded by a complex environment. This requires precisely capturing these features across various spatial scales. The third is the larger parameter size and difficulty in training and using it for real time application, which requires a smaller deep model favoring both training convergence and responding speed.

Therefore, the main goal of this research was to propose a novel deep learning model that integrates the techniques, such as residual learning, coordinate attention mechanism, and group convolution, into a deep multi-task learning (MTL) approach to simultaneously accomplish three disease detection tasks of identifying the infection site, classifying the infection stage, and estimating disease severity. This deep MTL model is expected to achieve higher detection efficiency compared with eight state-of-the-art deep learning models in terms of detection accuracy and parameter size. The key contributions of this research are as follows:

1) A deep multi-task learning (MTL) approach was developed to simultaneously accomplish three mummy berry disease detection tasks with limited data;
2) Novel superimposed attention mechanism modules applied to deep learning was found to enhance disease feature extraction from both channel and spatial perspectives, enabling better performance in an open and complex environment compared to other CNNs;
3) Integrating grouped convolution to MTL enabled it to learn a varied set of low-level and high-level disease features in a more parallel manner, resulting in a significant reduction in computational complexity and faster convergence.

## 2 Materials and methods

### 2.1 Overview

**Figure 1:**
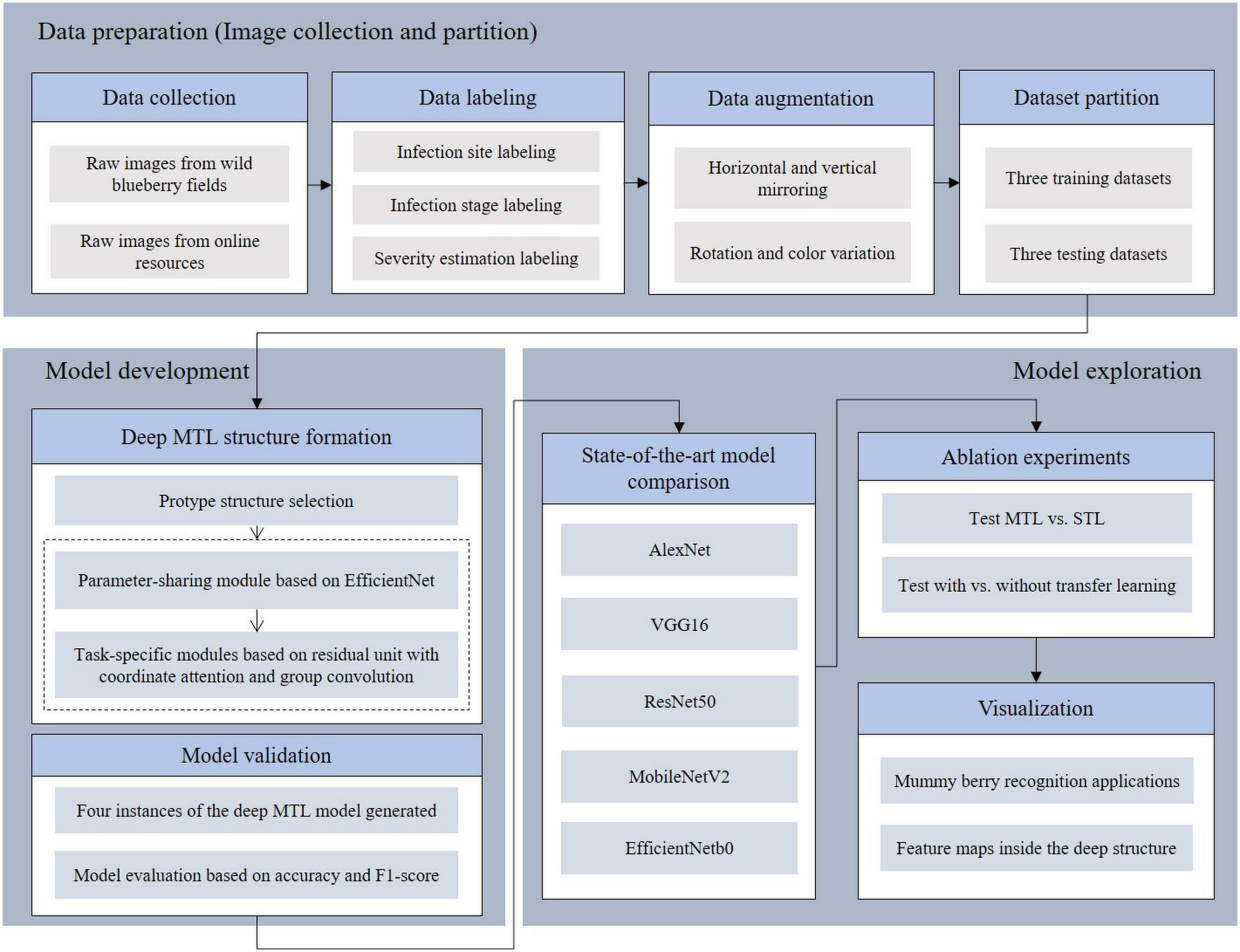
Overview of the three tasks of the mummy berry disease detection research: data preparation, model development and exploration.

This research investigated a deep learning based MTL framework to simultaneously identify infection sites (where diseased tissues are located), classify the infection stage (primary or secondary stage) and estimate the infection severity from raw images of lowbush blueberry stems. The task of building a deep MTL framework contains three main procedures represented as blocks, which are data preparation, model development and model exploration (**Figure 1**). In the data preparation block, healthy and diseased blueberry images were collected from fields and online resources and were manually labeled with the infection status. These labeled images were varied for the purpose of augmentation and were randomly distributed into three training datasets and three testing datasets. In the model development block, EfficientNet (Tan and Le, 2019) was used as the protype to establish the deep MTL framework having one parameter sharing module and three task-specific modules. Once the deep MTL framework (model) was formed, four instances of it with variations in parameters and configurations were generated, trained and tested. In the model exploration block, the four model instances were first compared with the state-of-the-art deep solutions in terms of accuracy and F1-score (see section 3.2 Evaluation metrics), then the advantages of using multi-task and transfer learning were determined by two sets of ablation experiments. Finally, several applications of mummy berry disease detection and feature maps of the deep MTL structure were visualized.

### 2.2. Preliminary

The life cycle of mummy berry disease contains two distinct stages. In the primary infection stage, successfully overwintered pseudosclerotia beneath fallen blueberry leaves start developing apothecia which discharge sexual spores (ascospores) in the early spring to infect leaves and flowers. Infected leaves often turn from greenish red or greenish pink color to a rosy brown and sometimes form a shepherd’s crook or curl. The infected emerging floral buds usually have a brown discoloration or blighted appearance (McGovern et al., 2012). Following primary infection, secondary asexual spores (conidia), appearing as a white to grey powder, are produced on the infected, blighted leaves and flowers. The production of asexual spores (conidia) is called sporulation and is regarded as the phase immediately preceding secondary infection, although for our modeling we included sporulation as the initial stage of secondary infection because it is the conidia which are vectored to the flower resulting in flower infection and subsequent fruit infection that result in secondary infection. Conidia are vectored by insect pollinators to open flowers, the conidia then germinate and grow down the style to reach and infect the ovules. Infected immature blueberry fruit initially appears waxy green, but begins to discolor as the disease develops. Finally, the infected mature berries become gray and shriveled while healthy mature berries are a waxy blue to purplish color. Secondary infection subsequently results in a mummified berry (Batra, 1983; Batra and Batra, 1985).

**Figure 2:**
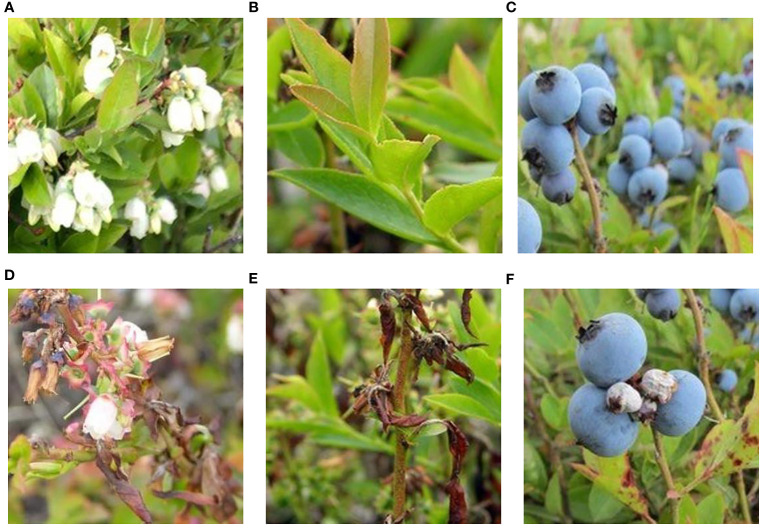
Examples of mummy berry infection site: healthy flowers **(A)**, healthy leaves **(B)**, healthy fruits **(C)**, infected flowers **(D)**, infected leaves **(E)** and infected (mummified) fruits **(F)**.

**Figure 3:**
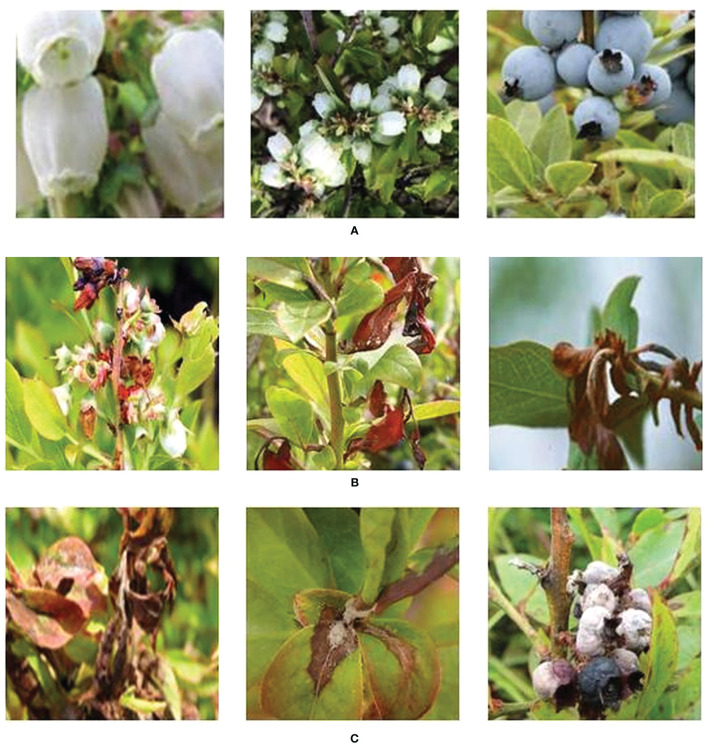
Examples of infection stage: healthy (row **(A)**, primary infection (row **(B)**, and sporulation on leaves initiating secondary infection, and secondary infection of mummified fruit (row **(C)**.

**Figure 4:**
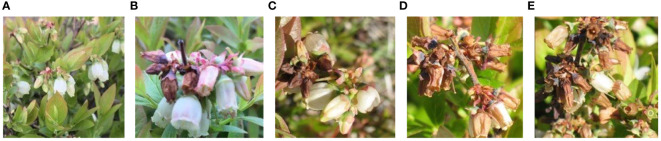
Examples of primary infection severity estimation: healthy **(A)**, very low **(B)**, low **(C)**, high **(D)**, very high **(E)**.

In reviewing the literature on the pathological characteristics of mummy berry disease, we emphasize that the symptoms occur only on leaves (primary infection), flowers (primary infection) and fruits (secondary infection) of lowbush blueberry plants. The color and shape of the infected plant organs are identifiable features that indicate the infection site. Since primary infection symptoms are only present in leaves and flowers, these can be distinguished by features such as white, powdery conidia on the sporulating organs just prior to secondary infection, and the mummified berries can act as a secondary infection symptom, so that the two infection stages can be classified from images. Also, the number of infected tissues and their occupied area on the image can provide useful information for a disease severity estimation. Therefore, our study site identification refers to the classification between healthy and diseased leaves, flowers and fruits (**Figure 2**). Stage classification is to distinguish infection phases by examining the symptoms that are exclusively featured by the primary or secondary infection stages (**Figure 3**). Severity estimation was performed by calculating the percentage of area occupied by the diseased tissues in relation to the whole image (**Figure 4**).

### 2.3 Dataset

#### 2.3.1 Data collection

The dataset created for this study contains raw images of healthy and diseased blueberry flowers, leaves and fruits. Our primary image source was research scientists at the University of Maine, Orono, Maine USA. Over the past several years, hundreds of lowbush blueberry images were acquired from the University of Maine’s lowbush blueberry experimental fields. These images were taken in a wild blueberry growing environment with a complex background. However, the number of raw blueberry images were still far less than the requirement of deep neural network training and validation. Therefore, we also used python Scrapy (www.google.com) to search and extract online mummy berry images, such as Google and the National Ecological Observatory Network (Bugwood.org) to expand our dataset. The extra images collected online not only helped to alleviate a data deficiency problem, but were useful for generalizing the training features of mummy berry disease, which is an effective way of reducing the risk of overfitting (Perez and Wang, 2017).

#### 2.3.2 Data labeling

A total of 927 raw blueberry images were collected which includes the categories of healthy and diseased flowers, leaves and fruits. The labeling process aimed at manually classifying each image to the corresponding categories according to its feature. For example, an image having the content of mummified berries was labeled as infected fruit and secondary infection stage. The labeling process was assisted by domain experts with their pathological expertise in mummy berry disease. Based on the obtained images, three datasets were generated to solve the three tasks of disease recognition. The three datasets were named as infection site identification dataset (**Figure 2**), infection stage classification dataset (**Figure 3**) and severity estimation dataset (**Figure 4**).

The infection site identification dataset consists of the images featuring healthy and diseased flowers, leaves and fruits, which was divided into 6 categories (**Figure 2**, **Table 1**); the infection stage classification dataset contains three categories of symptom images. The first is the healthy category that consists of healthy flowers, leaves and fruits. The second category is the primary infection stage involving infected pinkish leaves and emerging floral buds. The third category is the secondary infection stage featuring sporulating leaves and flowers, as well as mummified fruits (**Figure 3**, **Table 2**): the severity estimation dataset was created by calculating the percentage of area occupied by infected tissues in the whole image. This calculation was done by using an image segmentation method (Haralick and Shapiro, 1985) with manual corrections where necessary. For certain severity ranges, labels were assigned as follows: healthy (< 0.5%), very low (0.5% ~ 5%), low (5% ~ 10%), high (10% ~ 15%) and very high (>15%) (**Figure 4**, **Table 3**).

**Table 1:** The number of images in each of the six categories of the infection site identification dataset after data augmentation.

Infection site identification dataset			
Healthy flower	222	Infected flower	144
Healthy leaf	104	Infected leaf	90
Healthy fruit	172	Infected fruit	370
		Total	1102

**Table 2:** The number of images in each of the three categories of infection stage classification dataset after data augmentation.

Infection stage classification dataset			
Healthy	Primary infection	Secondary infection	Total
505	384	336	1225

**Table 3:** The number of images in each of the five categories of severity estimation dataset after data augmentation.

Severity estimation dataset					
Healthy	Very low	Low	High	Very high	Total
531	251	112	130	204	1228

#### 2.3.3 Data augmentation

**Figure 5:**
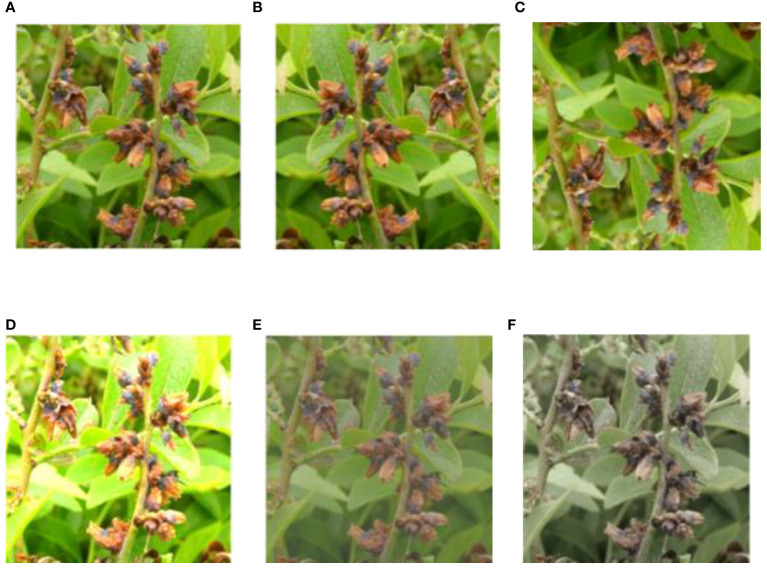
Example of data augmentation: original image **(A)**. Images **(B, C)** represent variants by flipping the original image horizontally and vertically, whereas images **(D-F)** represent variants by corrupting the original image in brightness, contrast and saturation, respectively.

Complex models particularly deep learning ones tend to suffer overfitting when trained with small narrowly constituted datasets (Shorten and Khoshgoftaar, 2019). To address this issue, data augmentation techniques were used to generate synthetic samples of the raw data in order to increase the generalization ability of the deep model (Perez and Wang, 2017). In this work, two distinct techniques of data augmentation were employed. The first data augmentation technique utilizes horizontal and vertical mirroring (**Figures 5B, C**) of the original images (**Figure 5A**). While the second data augmentation technique adjusts brightness, contrast, and saturation of the original images (Taylor and Nitschke, 2018) (**Figures 5D–F**). By doing this, the number of images in the original three-category dataset was increased. Considering the balance of data between categories, the total images in each of the three datasets were expanded to approximately 1200 images.

### 2.4 The model

#### 2.4.1 Theory of deep multi-task learning

One challenge of mummy berry disease detection is that the images of blueberry plants are usually a mixture of different plant parts with various visual focal distances and complex backgrounds. This challenge greatly restricts the conventional image segmentation methods for feature extraction and consequently causes a large uncertainty in recognition and classification. Deep learning architecture can take advantage of automated feature engineering to avoid the dependence on prior domain knowledge and human interventions in feature extraction. Convolutional neural networks (CNNs) are typical representations of deep learning architecture for image-based classification. The procedure of any CNN-based deep learning solution can be divided into two separated working phases, which are feature extraction and feature classification. The first phase takes raw images and outputs a feature vector to the second phase. Then the later processes the feature vector and outputs the result according to the specific classification requirements.

Another challenge using MTL is that compared with single task solutions, the accomplishment of multiple tasks (i.e., infection site identification, stage classification and severity estimation), may require larger training sample sizes, take more time to train, and experience higher risks of overfitting data. However, this problem can be solved if we sufficiently utilize the common knowledge among tasks instead of processing them separately, which leads to the multi-task leaning(MTL) concept in a deep learning architecture (Zhang and Yang, 2017). Multi-task Learning aims to take advantage of useful information obtained from multiple related tasks and by doing so, helps improve the generalization performance of all the tasks (Caruana, 1997). In order to fully characterize MTL, we provide a commonly accepted definition of MTL (Ruder, 2017).

#### 2.4.2 MTL Definition

**Figure 6:**
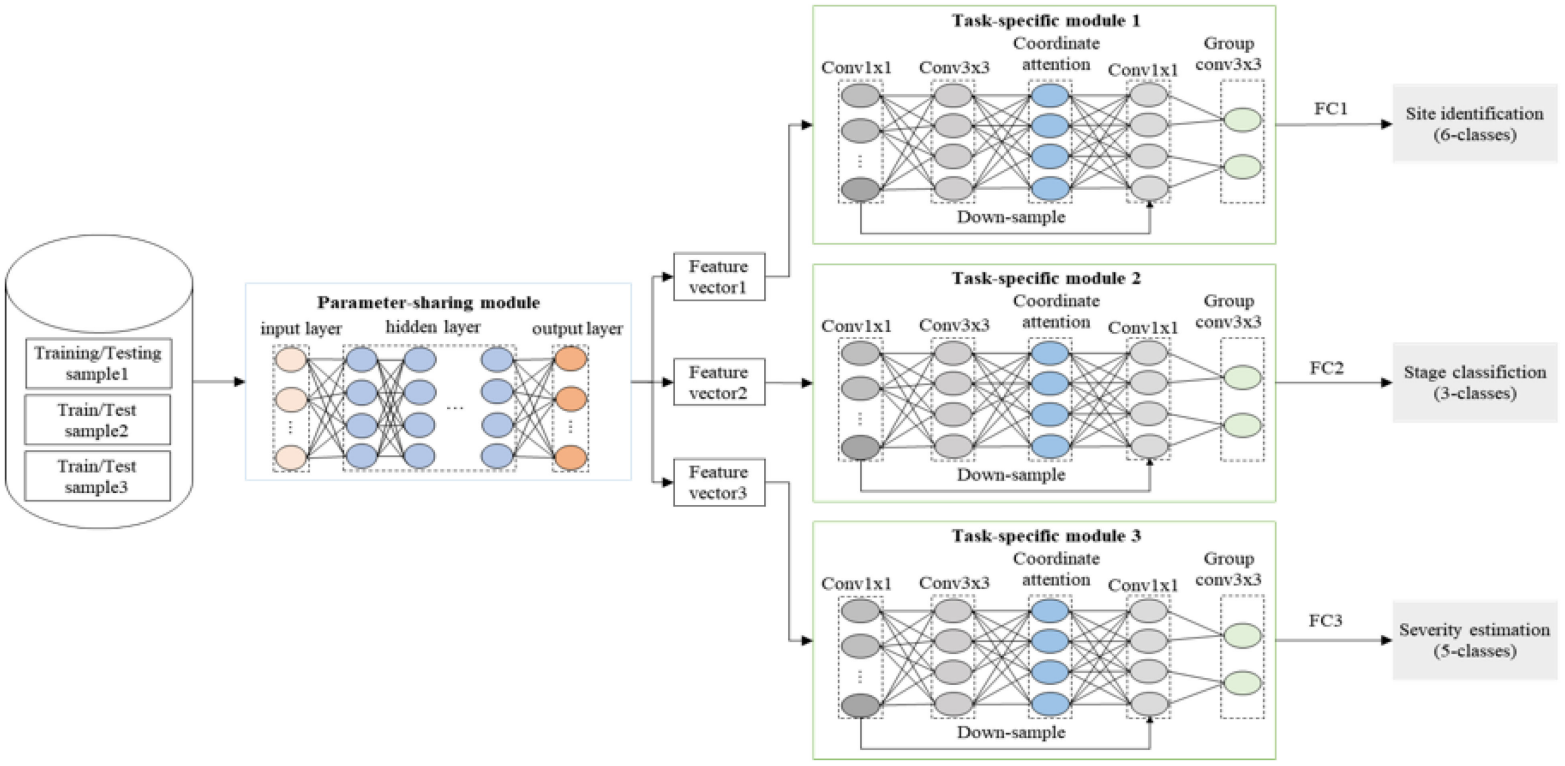
Architecture of our Deep MTL model, which is composed of a parameter-sharing module and three task-specific modules for simultaneously solving the three tasks (infection site identification, stage classification and severity estimation). FC stands for Full Connected Layer.

Given *m* learning tasks ${\left\{T_{i}\;\right\}}_{i=1}^{m}$ where all the tasks or a subset of them are related, multi-task learning aims to help improve the learning ability of a model for *T_i_* by using the knowledge contained in all or in part of the *m* tasks. Based on the definition of MTL, we focus on supervised learning tasks since images in this study were already precisely labeled. In the setting of supervised learning tasks, usually a task *T_i_* is accompanied by a training dataset *Di* consisting of *n_i_* training samples, i.e., $\;D_{i}=\;{\left\{X_{j}^{i},y_{j}^{i}\right\}}_{j=1}^{n_{i}}$ , where ${\text{X}}_{j}^{i}\in R^{d_{i}}$ is the *j^th^* training instance in *T_i_* and $y_{j}^{i}\;$ is its label. We denote ***X*** *_i_* as the training data matrix for *T_i_*, i.e., $X_{i}=\left(X_{1}^{i},\ldots ,X_{n_{i}}^{i}\right)$ . Here we consider a general setting for MTL that all the ***X*** *_i_* are different from each other. Therefore, our theoretical solution was that within a CNN architecture, firstly we employed a hard parameter-sharing technique (Caruana, 1993) to extract feature vectors for the three tasks because we hypothesized that many features are likely to be shared between images in the infection site, stage and severity datasets. For example, features indicating infection stage can be used to infer the site of infection, e.g., pixels of mummies can also be used to identify the secondary infection stage. Once the feature vectors were produced, we utilized the three task-specific deep structures to simultaneously perform site identification, stage classification and severity estimation. The theoretical solution is summarized in **Figure 6** (the technical details are given in the following implementation section).

#### 2.4.3 Deep MTL model implementation

The Deep MTL model for mummy berry disease recognition is composed of four modules: one Parameter-sharing module and three Task-specific modules **Figure 6**. The Parameter-sharing module is the most front basic module and takes raw pixels of plant images as input and automatically extracts features feeding the Task-specific modules. The Parameter-sharing module was constructed based on the basal deep structure of EfficientNet, which is a family of CNNs released in 2019 by Google AI (Tan and Le, 2019). We chose EfficientNet as the protype to implement the Parameter-sharing module of the Deep MTL because it offers excellent scaling ability in several dimensions such as network width, depth, and image resolution in either a simple or compound manner. This scaling ability allows EfficientNet to achieve ideal balance between accuracy and the size of network parameters.

Specifically, the Parameter-sharing module was made up of nine phase operations **Table 4**. We intentionally removed the final phase operation of the EfficientNet to compress the output channel. Therefore, the output channel of the Parameter-sharing module was compressed to 320 channels.

**Table 4:** The structure of the Parameter-sharing module based on the EfficientNet baseline network – Each row describes a phase *i* with *L_i_* layers, with input resolution <*H_i_*, *W_i_* > and output channels *C_i_* (Tan and Le, 2019).

Phase *i*	Operator *F_i_*	Resolution *H_i_* × *W_i_*	Channels *C_i_*	Layers *L_i_*
1	Conv3×3	224×224	32	1
2	MBConv1, K3×3	112×112	16	1
3	MBConv6, K3×3	112×112	24	2
4	MBConv6, K5×5	56×56	40	2
5	MBConv6, K3×3	28×28	80	3
6	MBConv6, K5×5	14×14	112	4
7	MBConv6, K5×5	14×14	192	5
8	MBConv6, K3×3	7×7	320	1
9	Conv1×1 & Pooling & FC	7×7	1280	1

The Task-specific module was constructed based on the residual unit (He et al., 2016) and featuring was performed with the attention mechanism (Hou et al., 2021) and the group convolution operation (Su et al., 2020) because successfully identifying fine features such as conidia on sporulating tissues is critical for infection stage classification. The Residual unit was employed to address the degradation problem and difficulties in learning identity maps for multiple non-linear layers, which have been proven effective in many visual tasks. Attention mechanisms (AM), used to “tell” a model ‘what’ and ‘where’ to attend, have been extensively studied (Mnih et al., 2014; Xu et al., 2015) and widely deployed for boosting the performance of modern deep neural networks. There are three typical AMs, i.e., The Squeeze-and-Excitation Networks (SE) (Hu et al., 2018) focuses on the attention information of the feature channels, the Convolutional Block Attention Module (CBAM) (Woo et al., 2018) uses both channel and spatial AM in a serial manner, and the Collaborative attention mechanism (CA) (Hou et al., 2021) employes both channel and spatial AM, but in a parallel manner.

In general, a visual attention mechanism can enhance information extraction from a channel or spatial perspective, or both. Channel-based AM boosts specific feature layers (i.e., channels) possessing more interesting information and lessens others in the feature map, while the spatial-based AMs can focus on specific interesting region of the feature space and ignore the background. The different characteristics of AM varieties and their successful applications indicate that the combination of these AMs in deep neural networks could be effective to solve this problem. Because focusing on features of interest across various spatial scale and abstract levels could be vital for extracting disease features from complex backgrounds with likely occlusions.

**Figure 7:**
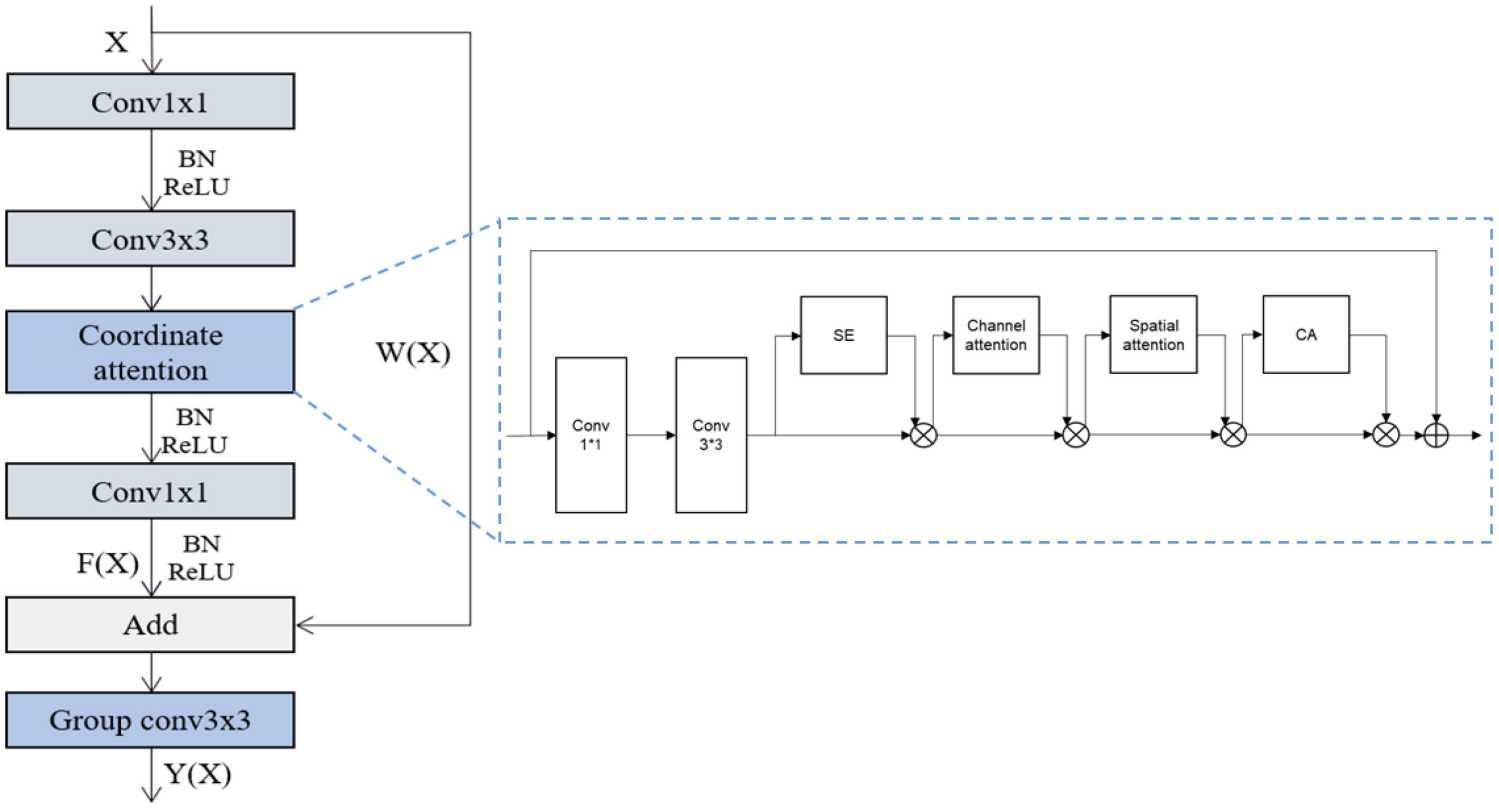
Structure of the Task-specific modules. Input comes from the feature vectors. The magnified part at the right detailed the structure of the attention mechanism module imbedded in the left structure. SE stands for the Squeeze-and-Excitation Networks, CBAM stands for the Convolutional Block Attention Module, and CA stands for the Collaborative attention mechanism.

We proposed a superposed structure combining the three attention mechanisms (SE, the CBAM, CA) in the CNN. After the convolutional layer, the features are learned in different channels by the SE attention mechanism, then by CBAM attention mechanism, learning through both the channel and space. Next, they are passed to the CA attention module to extract feature information as well as feature location information on the feature channels, and finally passed to the pooling layer and the fully connected layer. **Figure 7** delineates the structure of the Task-specific module.

The grouped convolution was also applied to use different sets of convolution filter groups on the same input image. It allows one to create two or more deep learning models that can be trained and backpropagated in a parallel manner (Su et al., 2020). In other words, this approach creates a deep network with a limited number of layers, so that they are replicated to form multiple pathways for convolutions on a single image.

The Parameter-sharing module and the three Task-specific modules formed the main structure of the Deep MTL model, which was named in this study as the MummyBerryNet. The workflow of MummyBerryNet is as follows. First, the raw images of the three tasks are alternately input into the Parameter-sharing module to obtain three feature vectors. Then, each feature vector is input into the corresponding Task-specific module, and finally the classification result is obtained through the fully connected layer. The architecture of MummyBerryNet is described in **Table 5**.

**Table 5:** The architecture of the Deep MTL model: MummyBerryNet.

Layer name	Output tensor	Configurations*
Input	3×224×224	Augmented images
Parameter-sharing module	320×7×7	EfficientNet
Task-specific module	2048×5×5	$\left[\begin{array}{c} 1\times 1,\;320\\ 3\times 3,\;640\\ 1\times 1,1280\\ 3\times 3,2048,C=32 \end{array}\right]\times 3$
FC1	6-classes	
FC2	3-classes	
FC3	5-classes	

*‘1×1′and ‘3×3′indicate the convolution operation with kernel equal 1 or 3; ‘C’ represents channel of convolution. ‘FC’ stands for Fully Connected Layer.

#### 2.4.4 Four instances of the deep MTL model

The baseline network, EfficientNet, is a family of eight versions of CNNs with different tradeoffs between performance and network size ranging from 5.3M to 66M parameters (Tan and Le, 2019). It provides a variety of options to achieve an expected accuracy with an affordable computational cost. Considering the uncertainty and complexity of mummy berry disease recognition, the exploration of the fitness of these models to the real and complex environment is worthwhile. Therefore, our solution was to choose four models to generate four instances of the Parameter-sharing module. The selection criterion used is as follows: we estimated the accuracy-cost ratio of eight versions and selected the top four as the candidate baseline networks (**Figure 1**. Model Size vs. ImageNet Accuracy of Tan and Le, 2019), which were EfficientNet-B0, B1, B2 and B3. Their parameter sizes were therefore compressed into the range between 5.3M and 12M. The four instances of the Deep MTL model, which was previously named as MummyBerryNet, were: MummyBerryNet-B0, MummyBerryNet-B1, MummyBerryNet-B2 and MummyBerryNet-B3. The four instances of MummyBerryNet were different in the Parameter-sharing module but were almost the same in the Task-specific modules, as shown in **Table 6**, **Tables A1-A4** (see **Appendix**). After model implementation, we then trained for and conducted experiments on each of the four instances independently.

**Table 6:** Configurations of the four instances of MummyBerryNet.

Input layer Model instances	Input	Parameter-sharing module	Task-specific Module^1^	FC1^2^	FC2	FC3
MummyBerryNet-B0	3×224×224	EfficientNet-B0, Output: 320×7×7 (**Table A1**)	$\left[\begin{array}{c} 1\times 1,\;320\\ 3\times 3,\;640\\ 1\times 1,1280\\ 3\times 3,2048,\\ C=32 \end{array}\right]\times 3$ Output: 2048×5×5	6 classes	3 classes	5 classes
MummyBerryNet-B1	3×240×240	EfficientNet-B1, Output: 320×8×8 (**Table A2**)	$\left[\begin{array}{c} 1\times 1,\;320\\ 3\times 3,\;640\\ 1\times 1,1280\\ 3\times 3,2048,\\ C=32 \end{array}\right]\times 3$ Output: 2048×6×6			
MummyBerryNet-B2	3×260×260	EfficientNet-B2, Output: 352×9×9. (**Table A3**)	$\left[\begin{array}{c} 1\times 1,\;320\\ 3\times 3,\;640\\ 1\times 1,1280\\ 3\times 3,2048,\\ C=32 \end{array}\right]\times 3$ Output: 2048×7×7			
MummyBerryNet-B3	3×300×300	EfficientNet-B3, Output:384×10×10 (**Table A4**)	$\left[\begin{array}{c} 1\times 1,\;320\\ 3\times 3,\;640\\ 1\times 1,1280\\ 3\times 3,2048,\\ C=32 \end{array}\right]\times 3$ Output: 2048×8×8			

^1^‘1×1′ and ‘3×3′ indicate the convolution operation with kernel equal 1 or 3 and ‘C’ represent channel of convolution. 2: ^2^ ‘FC’ stands for Fully Connected Layer.

#### 2.4.5 Training the deep MTL model

In the training process, we converted the input images (i.e., the training samples obtained from offline data augmentation, see subsection 2.3.3.) with the fixed size of 224 × 224 × 3 to meet the input size requirements of MummyBerryNet. The number of images in the training and testing datasets were kept at a 70/30 ratio. To train MummyBerryNet for the three mummy berry recognition tasks, several batches of computation were conducted. MummyBerryNet received new image samples from each new training batch. Each sample consists of images from all three tasks. The network weights were adjusted repeatedly until MummyBerryNet learned the most relevant discriminative features for a given task, i.e., the cross-entropy loss of the deep neural network converged. The training was performed by adapting pre-trained networks on the ImageNet dataset, which was achieved by means of transfer learning (details below). The four instances of MummyBerryNet were trained end-to-end, without freezing the training of any other layers. The stochastic gradient descent (SGD) algorithm was employed to improve the performance for all experiments. The learning rates were dynamically decreased by 1/10 at every 15 epochs during training, with the initial learning rate set at 0.005 for the first step. The weight decay of 0.0001 and batch size of 16 were used in the training process. The specifications of the optimizer and parameters for training MummyBerryNet are listed in **Table 7**. To implement this in Keras, we defined a step decay function and used *LearningRateScheduler* callback to take the step decay function as the argument and return the updated learning rates for use in the SGD optimizer.

**Table 7:** Specification of the optimizer and parameters for training MummyBerryNet.

Parameter	Setting
Optimizer	Stochastic Gradient Descent (SGD)
Loss function	Cross-Entropy
Learning rate*	0.005
Epochs	50
Batch size	16
Weight decay	0.0001
Momentum	0.9

∗ Decreased by a factor of 1/10 at every 15 epochs.

To make the training more efficient and achieve better performance of the CNN-based MummyBerryNet in the context of data limitation, the transfer learning technique was applied. Training was performed by adapting pre-trained networks on the ImageNet dataset. In this study, we only loaded the pre-trained weights of EfficientNet into the Parameter-sharing module of MummyBerryNet, while the weights of the Task-specific modules were initialized randomly. In particular, in order to apply the pre-trained weights to the Parameter-sharing module, we eliminated the weights of the ninth phase of the convolution and the fully connected layer.

## 3 Experiments

### 3.1 Experimental setup

In order to evaluate the efficacy of our model in solving the three tasks of mummy berry disease detection, the performance of training and testing the CNN-based MummyBerryNet was first evaluated by widely accepted metrics (see below), then three model exploration experiments were conducted. They were: 1) the performance comparisons between the state-of-the-art deep learning models; 2) the ablation experiments for testing the effects of MTL scheme and transfer learning; and 3) the visualization for several mummy berry disease detection applications. Ablation experiments are used to study the performance of an AI (artificial intelligence) system by removing certain components, to understand the contribution of the component to the overall system.

The deep learning frameworks used for performance comparisons were AlexNet, VGG16, ResNet50, MobileNetV2, and EfficientNet. The selection of these six deep learning models was motivated by the fact that, except for EfficientNet, the other eight models (AlexNet, VGG16, ResNet50, MobileNetV2, EfficientNet-B0, EfficientNet-B1, EfficientNet-B2 and EfficientNet-B3) have established themselves as the most renowned and widely used CNNs for image classification tasks. These CNNs are widely used as benchmarks for evaluating deep learning models (Chen et al., 2023). MummyBerryNet was compared with the six state-of-the-art deep learning frameworks under the same experimental configuration conditions. In the experimental process, we found that the initial learning rate had a strong influence on the performance of all models. By conducting multiple experiments on a training set, we determined the optimal learning rates for six models ranged from 0.001 to 0.005. In contrast with the transfer learning process of MummyBerryNet, which was given previously, we first loaded all the pre-training weights for the six state-of-the-art models, and then modified the number of output features of the fully connected layer to train them.

The ablation experiment had two purposes: 1) evaluate the impact of transfer learning on model performance in contrast with the scenario where no transfer learning was applied; and 2) test the advantages of knowledge sharing for multiple-task learning. Therefore, we disintegrated the Deep MTL model into several STL modes, trained them and then compared them with the original MTL model. The experimental configuration of the two ablation experiments was basically the same as that in the comparison experiment.

All experiments were conducted based on the publicly available code of PyTorch (Machine learning open-source library) framework and a CPU/GPU platform which was built with a Xeon(R) 2.20 GHz (E5-2650 v4) CPU, 128 GB of memory and one Tesla P100-PCIE-12GB Graphics board.

### 3.2 Evaluation metrics

To estimate the effectiveness of MummyBerryNet in solving the MTL problem of mummy berry disease detection, the metric of accuracy was used. Specifically, the metric of accuracy is defined as the proportion of true results (including both the True Positives and the True Negatives) among the total number of samples examined (Equation 1):


(1) $$Accuracy=\frac{TP\;+\;TN}{TP\;+TN\;+FP\;+\;FN}$$

where TP=True Positive, FP=False Positive, TN =True Negative, and FN=False Negative rates.

However, accuracy carries more weight on the True Positives and True Negatives than the False Positives and Negatives. This may bias perception of the disease detection results. Furthermore, accuracy has been found to be sensitive to imbalanced samples.

To overcome this problem, we added the F1-score to balance the evaluation metrics, which gives more weight to False Negatives and False Positives, and also performs better when the sample classes are imbalanced (Qu and Liu, 2020). The F1-score is defined as (Equations 2–4):


(2) $$F1-score=\frac{2\;*\;Precision\;*\;Recall}{Precision+Recall}$$

where,


(3) $$Precision=\frac{TP}{TP+FP}$$

(4) $$Recall=\frac{TP}{TP+FN}\;$$

The third metric used was certainty, which was used for mummy berry disease detection applications. For each run of the MummyBerryNet model, the output is a vector containing the likelihood that the detection result should be classified to a category. The category with the maximum likelihood or certainty value is then regarded as the prediction result of the model. We applied the Softmax function (Liu et al., 2016) to an *n*-dimensional vector of the model output and rescaled them so that the elements of the *n*-dimension were in the range [0,1] and summed to 1. The output of the Softmax function is defined as the detection certainty of an input image (Equation 5):


(5) $$Softmax\left(X_{i}\right)=\frac{exp\left(X_{i}\right)}{\sum_{j}exp\left(X_{j}\right)}$$

where *X* represents the n-dimensional vector outputted by MummyBerryNet, *X_i_* represents a component of *X*, $\sum_{j}\;\text{exp}\;\left(X_{j}\right)$ represents the sum of *X*.

## 4 Results

### 4.1 Model training and validation

**Figure 8:**
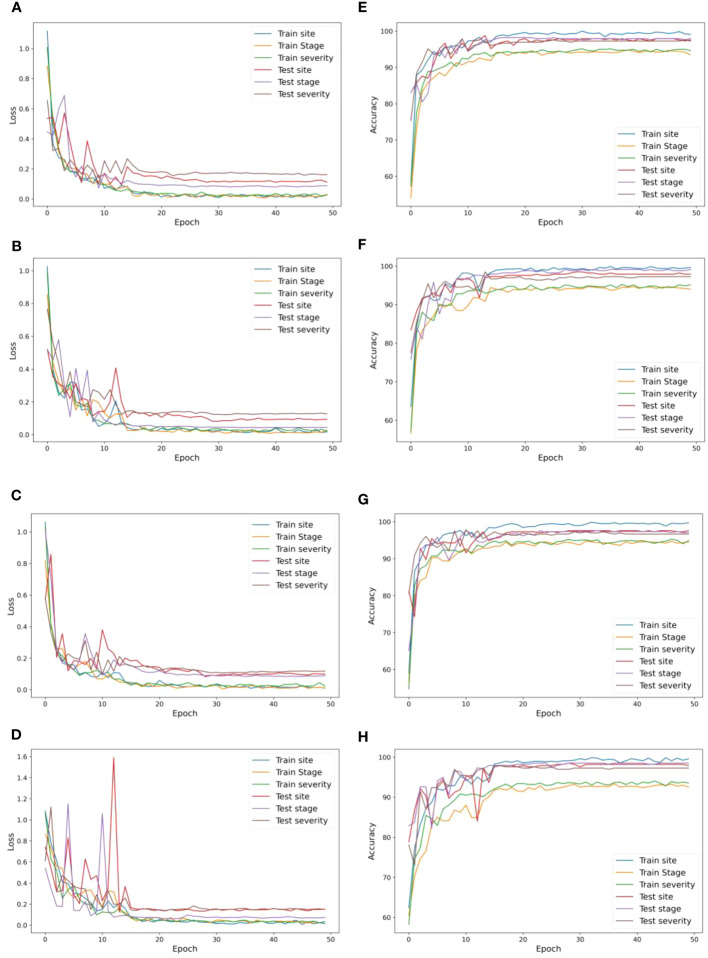
The classification cross entropy loss (left) and detection accuracy (right, in percent) during model training and validation. Panels **(A–D)** were: the model loss changing with training and testing iterations of the four instances MummyBerryNet-B0 to B3, respectively; and panels **(E–H)** were the detection accuracy changing with training and testing iterations of the four instances: MummyBerryNet-B0 to B3.

After inspection of the dynamics of cross entropy loss and accuracy in both the training and validation process of the four instances of MummyBerryNet, we found that the well trained MummyBerryNet is a good-fit model for mummy berry disease detection. Overall, the training loss decreased very quickly before epoch 10, then continuously but slowly went down and started to converge after epoch 30, suggesting an effective training process **Figure 8**. The differences of cross entropy loss between training and validation were less than 5%, which demonstrated that MummyBerryNet can learn the fundamental patterns of mummy berry disease in the training samples and had excellent generalizability across different datasets without overlapping. Our results also showed that MummyBerryNet’s risk of overfitting is fairly low. As for accuracy, MummyBerryNet also quickly reached the best performance zone, higher than 95%, after epoch 15. The overall speed of convergence of the four instances of MummyBerryNet was better than our expectation, which can be explained by the contribution of transfer learning and weight sharing among tasks. The total number of parameters (Parameter-sharing plus Task-specific modules) of the four instances of MummyBerryNet ranged from 21.4M to 28.4M (see **Table A5 in Appendix A**). The four instances were only 30% of the size of the biggest NefficientNet version (B7).

All four instances of the MummyBerryNet model achieved high accuracy and F1-scores **Table 8** in mummy berry disease detection. In the infection site identification task, the first instance, i.e., MummyBerryNet-B0 had the highest accuracy (96.81%) and F1-score (97.03%) and the lowest record was obtained by MummyBerryNet-B2 with an accuracy of 95.63% and F1-score of 95.50%. In the infection stage classification task, MummyBerryNet-B1 achieved the highest accuracy of 97.13% and the highest F1-score of 97.68%, while the lowest accuracy and F1-score were recorded by MummyBerryNet-B2. The second instance MummyBerryNet-B1 also achieved the best accuracy of 96.51% and best F1-score of 92.04% in the severity estimation task, which can be regarded as the best candidate of the four instances of our Deep MTL model, if we also take into consideration that it had the second smallest parameter size and the second lowest computational cost (see **Appendix A, Table A5**).

**Table 8:** Performance^1^ of the four instances of MummyBerryNet on the validation dataset.

Instance of the Model	Site identification		Stage classification		Severity estimation	
	Accuracy	F1-score	Accuracy	F1-score	Accuracy	F1-score
MummyBerryNet-B0	**96.81**	**97.03**	96.26	96.83	95.91	92.31
MummyBerryNet-B1	96.52	96.56	**97.13**	**97.68**	**96.51**	**92.04**
MummyBerryNet-B2	95.63	95.50	95.68	96.25	95.91	92.14
MummyBerryNet-B3	96.52	96.72	96.55	97.04	95.91	91.12

^1^The best model performance is shown in bold.

**Figure 9:**
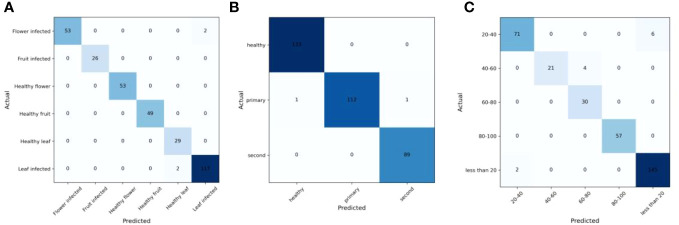
Confusion matrix for MummyBerryNet model for three tasks: the infection site identification task **(A)**; the infection stage classification task **(B)**; and the severity estimation task **(C)**.

A Confusion matrix is an important statistical tool used for machine learning model analysis to evaluate the performance of a classification task. It represents the relationship between the predicted results and the true labels generated by the model. This matrix is a tabular representation that displays the count of accurate and inaccurate predictions made by the model through a comparison of predicted values with the actual values **Figure 9**. The elements on the diagonal represent the count of samples correctly predicted by the model, whereas the off-diagonal elements represent misclassifications. In multi-class classification tasks, the accuracy of the model for each category can be accurately calculated by analyzing the diagonal elements.

### 4.2 Comparisons

**Figure 10:**
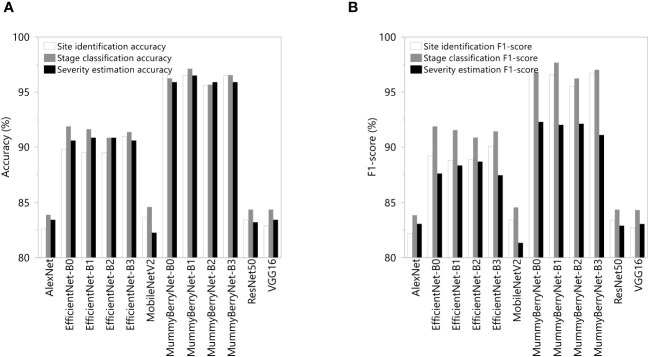
Comparations of detection accuracy **(A)** and F1-score **(B)** between the four instances of MummyBerryNet and the eight state-of-the-art CNNs in the three detection tasks: the infection site identification; the stage classification; and the severity estimation.

The systematic comparisons between the four instances of MummyBerryNet model and the eight state-of-the-art CNNs demonstrated that the MummyBerryNet model outperformed the eight CNNs (**Figure 10**; data can be found in the **Appendix B, Table B2**). In the site identification task, MummyBerryNet-B0, B1 and B3 achieved the highest accuracy and F1-score, while the AlexNet and MobileNetV2 were the lowest detectors, which were around 14% lower than our model. Even the worst instance of our model (MummyBerryNet-B2) outperforms the other comparison models in terms of both accuracy and F1 scores. In the stage classification task, all four instances of MummyBerryNet were listed as the top-4 best competitors on both accuracy and F1-score. MummyBerryNet-B1, as the best detector, was 4.5% higher in accuracy and 5% higher in F1-score than EfficientNet-B2. In the severity estimation task MummyBerryNet-B1 overwhelmingly outperformed the other CNNs. Overall, MummyBerryNet had obvious advantages in site identification and stage classification compared to the eight CNNs, but the advantages declined around 1.5% in the severity estimation task. Due to the complex background and high mixture of plant parts, as well as noise and distortion, all methods faced a great challenge in estimating the proportion of diseased plant tissues perfectly.

### 4.3 Ablation experiments

**Figure 11:**
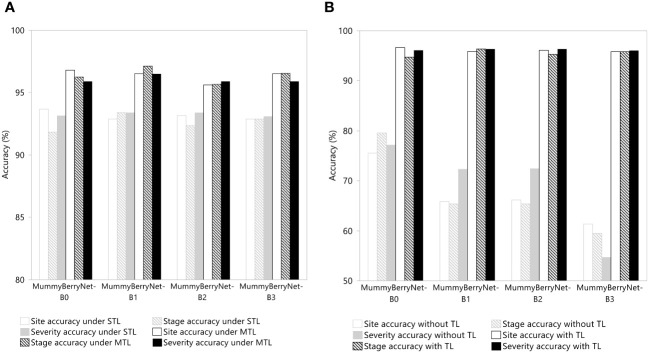
Efficacy of MTL **(A)** and transfer learning (TL) **(B)** in network performance enhancement in the three detection tasks: the infection site identification; the stage classification; and the severity estimation of the four instances of MummyBerryNet.

The results of the ablation experiments demonstrated the effectiveness of applying MTL and transfer learning. In the first ablation experiment, two scenarios were compared to determine differences between MTL and STL learning in mummy berry disease detection (**Figure 11A**, data in **Appendix B, Table B3**). On average, MTL can increase detection accuracy by 3% compared to the STL learning scheme. However, the advantage gained by MTL was dominated by stage classification, which was 6% higher than the STL scheme and was twice as high as that in severity estimation. This result revealed that the advantage of using parameter or knowledge sharing between different tasks might be negatively correlated to the amount of information in the training samples because the disease severity needs much more information per unit to be accurately estimated than the other two categories, even when we had balanced the samples for the three tasks for training.

In the second ablation experiment, two scenarios in which model trainings with or without adopting transfer learning were compared. As shown in **Figure 11B** (data in **Appendix B, Table B4**), transfer learning can increase disease detection accuracy up to 30%, a large increase that once again demonstrated the effectiveness of transfer learning. Almost all of the four instances of MummyBerryNet gained the same level of enhancement by using transfer learning.

### 4.4 Visualization

#### 4.4.1 Applications

**Figure 12:**
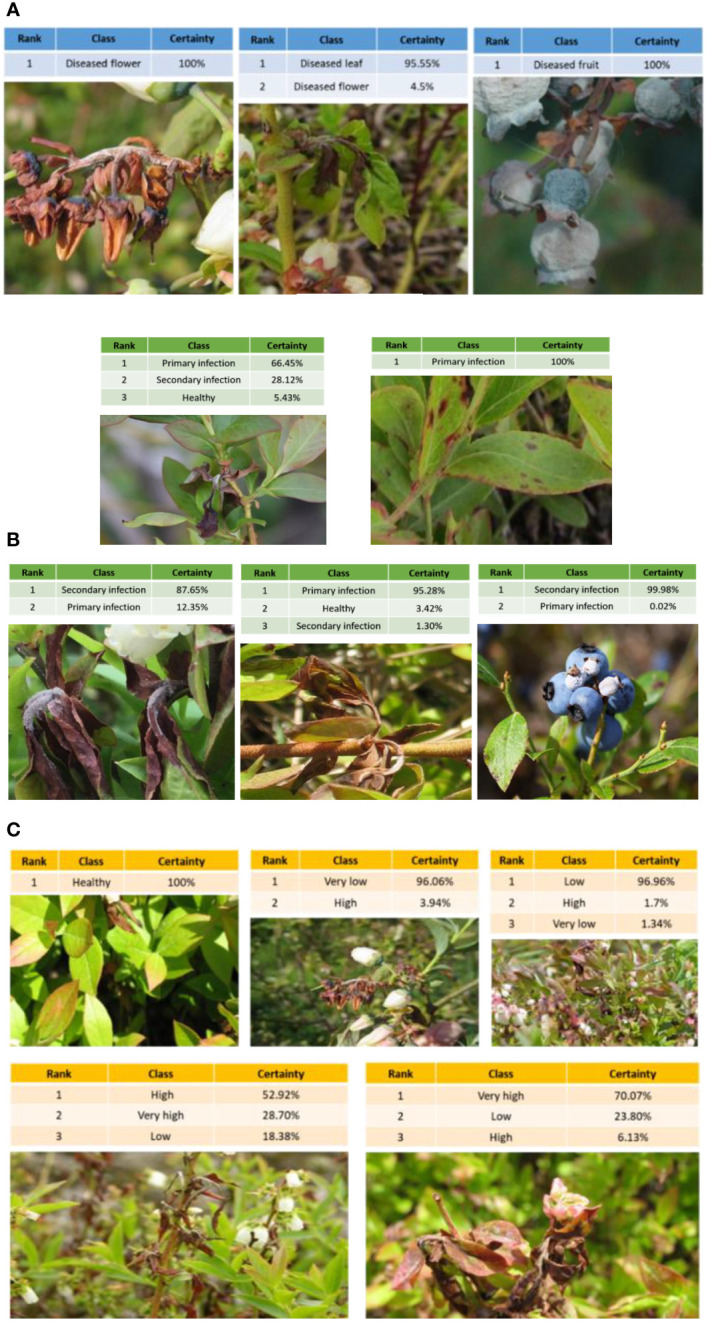
Applications of infection site identification **(A)**, stage classification **(B)** and severity estimation **(C)** using MummyBerryNet-B1, the best detector among the four instances of MummyBerryNet model. In A, the right picture in the second row is an example of a false positive identifying a picture of another leaf spot as primary infection by mummy berry.

MummyBerryNet-B1, having the optimal tradeoff between accuracy and computational cost among the four instances of MummyBerryNet, was selected as the best detector and applied to three mummy berry disease detection tasks. **Figure 12** visualized the detection results for infection site identification (a), stage classification (b) and severity estimation (c), respectively. In each task, a randomly selected image was input into MummyBerryNet-B1. The detection tasks were then calculated, and the detection result was listed in tables. Each table had three columns indicating the ranking of detection probability of each predicted class and certainty. In the middle panel of **Figure 12A**, for example, both the likelihood of diseased leaf and flower in the image were detected with 95.55% and 4.50% certainty for leaf and flower, respectively. The detected classes were listed and sorted on descending certainty, while the classes with zero likelihood were removed from the table.

#### 4.4.2 Feature map inside the deep structure

**Figure 13:**
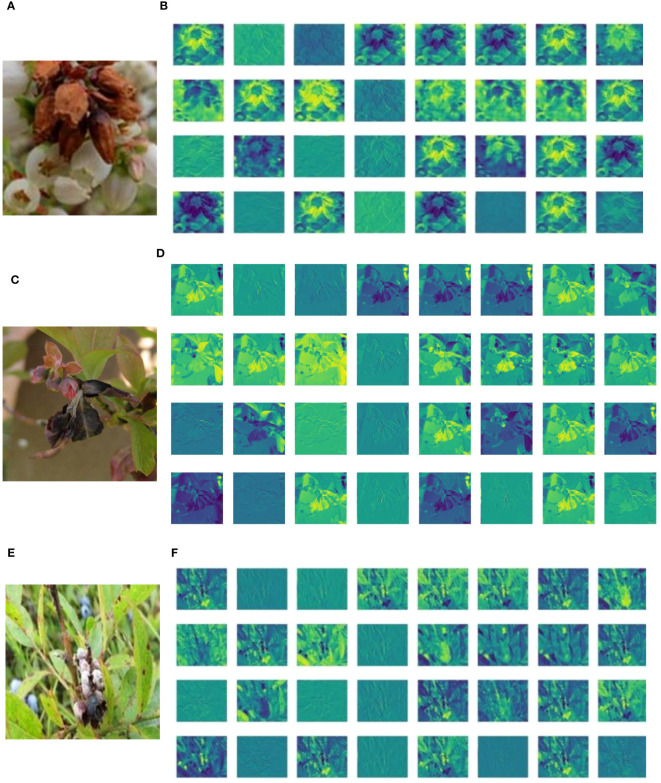
Visualization of CNNs (MummyBerryNet) in the initial layers, which consist of a 3x3 convolution and batch normalization. Panels **(A, C, E)** represent the original images (size 224× 224) of blueberry flowers, leaves, and fruits, respectively; panels **(B, D, F)** were the feature maps of flowers, leaves, and fruits with training (size 112 × 112).

**Figure 14:**
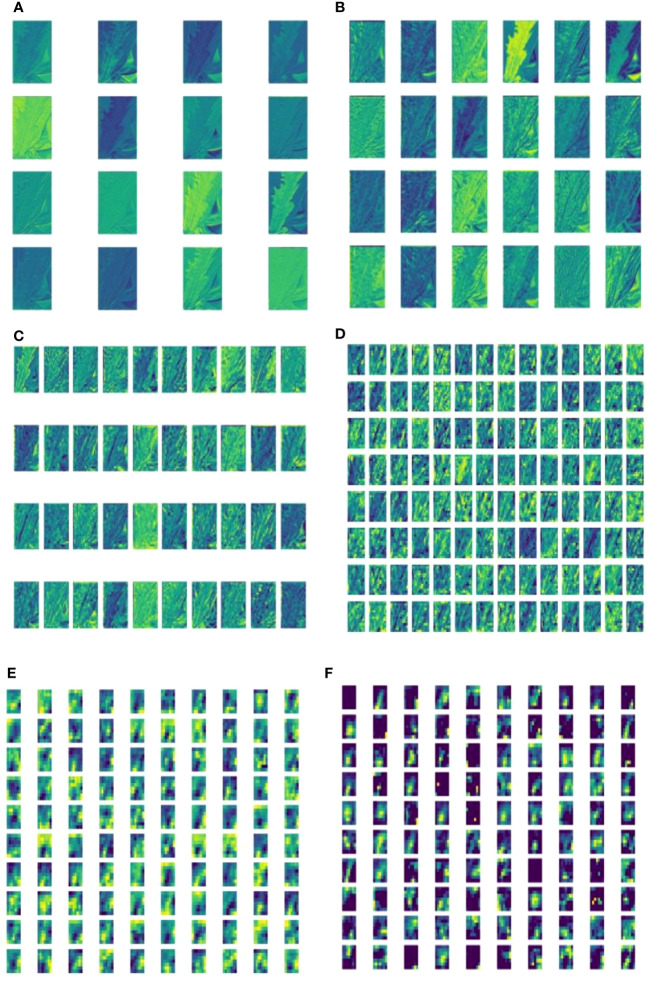
Visualization of feature maps inside MummyBerryNet. Panel images **(A-E)** were obtained by the Parameter-sharing module (layer), Panel **(F)** images were obtained by the Task-specific module (layer).

Deep feature visualization of CNNs can help us to understand the process of feature extraction from the millions of image patches. This is also useful to adjust the optimal hyperparameters during training. The visualization results of deep learning shallow convolutional neural network conform to the image information understandable by humans using visual perceptions. This helps us to intuitively observe and understand the focus area of model feature extraction in specific disease detection tasks. A feature visualization was constructed to observe the correspondence behavior of the first convolution layer of MummyBerryNet in **Figure 13**. The visualization demonstrated that feature maps obtained from the first convolution layer primarily focused on the color and contour extraction of blueberry flowers, leaves and fruits, which can clearly show that the lesion areas of mummy berry diseases received special attention and better explained what our model had actually learned. In order to present the transformation of feature extraction in MummyBerryNet, **Figure 14** illustrates the comparisons between different feature maps obtained by the layers in the Parameter-sharing and Task-specific module. In the feature maps, each point was a rectified activation, meaning that the brighter it was, the greater activation value it represented. The difference between modules and layers clearly demonstrated that MummyBerryNet was characterized by an excellent learning process for fine grain aspects of blueberry plant tissue with disease.

## 5 Discussion

### 5.1 Detection of mummy berry disease under a complex background

This study proposed a deep learning-based computer vision solution for automatic mummy berry disease detection. To the best of our knowledge, this is the first method in the lowbush blueberry research community that can automatically and simultaneously identify infection site, classify infection stage, and estimate severity from field acquired lowbush blueberry plant images. The primary advantage of this machine learning approach is that a Convolutional Neural Network (CNN) was employed to detect various diseased plant parts interacting with a complex background, but no explicit feature engineering was involved (Liu et al., 2020). Although automatic plant disease detection using deep neural networks is no longer a “cutting-edge” technique, detecting small lesions mixed with different types of noise and coexisting plant parts varying in size, shape, angle, focal distance, and contrast is still a big challenge (Liu and Wang, 2021), it can be solved by the integrated attention mechanism. In general, a visual attention mechanism can enhance information extraction from a channel or spatial perspective, or both. A channel-based attention mechanism boosts specific feature layers (i.e., channels) possessing more interesting information and lessens others in the feature map, while the spatial-based attention mechanisms can focus on a specific region of the feature space and ignore the background. The different characteristics of attention mechanism varieties and their successful applications indicated that the combination of attention mechanisms in deep neural networks is effective to solve this problem. Focusing on features of interest across various spatial scale and abstract levels is vital for extracting disease features from a complex background with likely occlusions, which has been proven by our experimental results.

In addition, we used a unique deep structure design with the help of visualization to understand how the focus area of model feature extraction operates for specific disease detection tasks (**Figures C1**, **C2**). This approach is particularly effective when dealing with small and low contrast targets. This is because visualization can help to identify different traits such as color, shape and contour in different layers (Nagasubramanian et al., 2019) (**Figure 14**). Furthermore, our four-instance model exploration method also greatly helped to refine model structure for mummy berry disease detection (Picon et al., 2019). In comparison with a similar technical approach in which a depth-separable CNN was employed instead of a standard convolution to detect grape leaf disease (Liu et al., 2020), our method performed better in detection accuracy even if our counterpart already outperformed the standard ResNet and GoogleNet structure (Thangaraj et al., 2021).

When it comes to plant disease detection in a complex background, our method has achieved superior detection accuracy in contrast with the state-of-the-art CNN-based counterparts (Xie et al., 2020; Chen et al., 2022; Liu and Zhang, 2022). The most important explanation is the application of grouped convolutions. Taking one modular block of a filter group and replicating it allowed us to build wider networks so that the learned features can be largely diversified. With the help of grouped convolution, our method is able to learn a varied set of low-level and high-level features, which is vital to more accurately detect disease in real farming conditions. In addition, the data augmentation employed in this work is able to capture and add some extra hidden features in the original data set, which is particularly useful in the context of a complex background where multi-level features are usually needed (Barbedo, 2018). Our method has shown better practicability when compared with recent advances in deep learning-based plant disease recognition solutions, such as the plant disease classification systems developed by Ferentinos (2018) and Mohanty et al. (2016) where lesions were finely focused and the target plant organs were imaged in a laboratory model with high resolution, simple uniform background and no overlap among plant organs and other tissues. In the real farming environment, lighting issues and occlusion problems are common challenges that have to be considered. In comparison with current advancements (Martinelli et al., 2015; Liu and Wang, 2020a; b), our deep MTL method with shared feature capturing has shown an alternative way to overcome complex and chaotic background issues.

### 5.2 Multi-task learning with lower computational cost and higher training efficiency

The second advantage or enhancement in our model is that the MTL scheme combined with transfer learning has shown a promising solution for image-based agriculture disease detection with lower computational cost and higher training efficiency. This advantage came from the parameter sharing mechanism. Our unique model structure, one Parameter-sharing module plus three Task-specific modules in MummyBerryNet, not only can take advantage of correlated knowledge representations among tasks to effectively decrease the risk of overfitting (Caruana, 1997), but also can train multiple tasks all together to implicitly decrease the required training samples as well as training time. The hypothesis that many features are shared between images in the infection site, stage and severity datasets therefore has successfully been tested. One example is that the pixels of mummies can be used to identify both the infection site and the secondary infection stage.

The most successful instance of the MummyBerryNet model, i.e. MummyBerryNet-B1, achieved the highest accuracy and the highest F1-score in both stage classification and severity estimation, but only had one-sixth of the network parameter size (23.98M vs. 138.36M) and one-fifteenth of the computational cost (1.13G vs. 15.48G of FLOPs, see **Appendix A, Table A5**) compared with the most popular CNN: VGG16. This advantage can be even larger if transfer learning is applied. One surprising finding in our ablation experiments was that transfer learning can significantly boost the accuracy of severity estimates (up to 30 percent on average). Since the estimation of disease severity requires much more information than classifying diseased plant parts and stages, such as differences in size, density, color and scale to make a reasonable estimation, this feature also made our solution very promising for future mobile deployment such as a drone carried task unit for real-time field surveillance.

The grouped convolutions imbedded into the MTL paradigm is also an important reason for performance superiority. In this approach, each filter convolves only on some of the feature maps obtained from kernel filters in its filter group, resulting in less redundant convolutions. It allows us to drastically lower the computations to get output feature maps. It also enables efficient data and model parallelism, which obviously benefits faster convergence, compared to the methods proposed by Chen et al. (2022) and Liu and Zhang (2022).

### 5.3 Limitations and future research direction

One of the limitations of MummyBerryNet is that we found all of its four instances had difficulty in detection of sporulation on shoots. One example was that in the mummy berry disease detection application (the first image in the B section of **Figure 12**), sporulating tissues had a higher probability of misclassification than other categories, e.g., the certainty of secondary infection was only 87.65%. This was due to the very limited samples of sporulation, which cannot train the deep structure well enough for the generality necessary for the task of accurate detection. This made the detection probability even lower when facing the small size and low contrast feature of sporulating tissues. We may need more relevant training data and particular attention to the mechanism (Fukui et al., 2019) of deep learning in the future version of MummyBerryNet to improve the detection accuracy. The second limitation was difficulty in the estimation of infection severity. We found that the accuracy of severity estimation was always the lowest among the three tasks (**Figures 10**, **11**). It is no doubt that sophisticated image segmentation with a complex background decreased the accuracy of severity estimation. However, we still believe that the primary reason was the manual labeling of severity levels as the function of infected area. Manually labeling severity levels doesn’t provide enough information connecting depth and scale of overlapped organs to a severity level. In future research, multi-view approaches (Zhou et al., 2021) integrating scale-aware information (Zhang et al., 2017) could be a feasible way to tackle this issue.

Aside from the above objective limitations, the technical limitation is about how multiple attention mechanisms could be effectively applied and collaborated with CNN. Although our experimental results suggested that the use of channel and spatial AMs are effective there is a need for further tuning to better facilitate real disease detection applications. In our current model, superimposing three different attention mechanisms in the residual blocks, may not be the best solution in terms of accuracy and performance. The superimposed attention mechanisms may be redundant in the feature extraction process by repeatedly extracting image features on different channels and spaces, leading to unexplainable results. However, due to the limitation of interpretability of deep learning, it is still unclear how these channel or spatial AMs should be combined and how the combination of AMs should be incorporated into the deep neural networks to effectively detect sporulation on shoots. The type of AMs, the number of AMs and their positions in the deep neural network need to be systematically investigated in future research. Due to the limited resources, this research was not able to conduct this investigation. But it is important to examine this uncertainty of AM corporation in tiny feature detection in an open and complex environment.

## 6 Conclusion

To solve the practical problem of field disease surveillance, this research proposed an innovative multi-task learning mode for mummy berry disease diagnosis. The model integrated the techniques, such as residual learning, coordinate attention mechanism, and group convolution, into a deep multi-task learning (MTL) approach to simultaneously accomplish three disease detection tasks of identifying infection sites, classifying infection stages, and estimating disease severity. This deep MTL model achieved higher detection efficiency compared with the state-of-the-art deep learning models in terms of detection accuracy and performance, due to the three key contributions: 1) the MTL approach can simultaneously accomplish three mummy berry disease detection tasks with limited data; 2) A novel superimposed attention mechanism modules applied to deep learning can enhance disease feature extraction from both channel and spatial perspective, enabling better performance in open and complex environment compared to other CNNs; and 3) Integrating grouped convolution to MTL enables to learn a varied set of low-level and high-level disease features in a more parallelism manner, resulting a significant lower computational complexity and faster convergence. These features make our solution very promising for future mobile deployment such as a drone carried task unit for real-time field surveillance. As an automatic approach to fast disease diagnosis, it can be a useful technical tool to provide growers real time disease information that can prevent further disease transmission and more severe effects on yield due to fruit mummification.

## Data availability statement

The raw data supporting the conclusions of this article will be made available by the authors, without undue reservation.

## Author contributions

HQ: Conceptualization, Funding acquisition, Investigation, Methodology, Project administration, Writing – original draft. CZ: Data curation, Investigation, Software, Validation, Writing – original draft. HJ: Data curation, Investigation, Software, Validation, Visualization, Writing – original draft. RH: Data curation, Formal Analysis, Investigation, Methodology, Visualization, Writing – original draft. DW: Conceptualization, Funding acquisition, Project administration, Resources, Writing – review & editing. SA: Conceptualization, Data curation, Resources, Supervision, Validation, Writing – review & editing. FD: Formal Analysis, Investigation, Resources, Supervision, Validation, Writing – review & editing.

## References

[B1] Alom M. Z. Taha T. M. Yakopcic C. Westberg S. Sidike P. Nasrin M. S.. (2019). A state-of-the-art survey on deep learning theory and architectures. Electronics 8, 292. doi: 10.3390/electronics8030292

[B2] Alruily M. (2021). Classification of arabic tweets: A review. Electronics 10, 1143. doi: 10.3390/electronics10101143

[B3] Barbedo J. G. A. (2018). Impact of dataset size and variety on the effectiveness of deep learning and transfer learning for plant disease classification. Comput. Electron. Agric. 153, 46–53. doi: 10.1016/j.compag.2018.08.013

[B4] Batra L. R. (1983). Monilinia vaccinii-corymbosi (Sclerotiniaceae): Its biology on blueberry and comparison with related species. Mycologia 75, 131–152. doi: 10.1080/00275514.1983.12021642

[B5] Batra L. R. Batra S. W. T. (1985). Floral mimicry induced by mummy-berry fungus exploits host’s pollinators as vectors. Science 228, 1011–1013. doi: 10.1126/science.228.4702.1011 17797664

[B6] Bock C. H. Poole G. H. Parker P. E. Gottwald T. R. (2010). Plant disease severity estimated visually, by digital photography and image analysis, and by hyperspectral imaging. Crit. Rev. Plant Sci. 29, 59–107. doi: 10.1080/07352681003617285

[B7] Caruana R. (1993). Multitask learning: A knowledge-based source of inductive bias1. In: Proceedings of the Tenth International Conference on Machine Learning; 1993 Jun 27-29; Citeseer, Amherst, MA, USA, p. 41–48. doi: 10.1016/b978-1-55860-307-3.50012-5

[B8] Caruana R. (1997). Multitask learning. Mach. Learn. 28, 41–75. doi: 10.1023/A:1007379606734

[B9] Chen R. Qi H. Liang Y. Yang M. (2022). Identification of plant leaf diseases by deep learning based on channel attention and channel pruning. Front. Plant Sci. 13, 1023515. doi: 10.3389/fpls.2022.1023515 36438120 PMC9686387

[B10] Chen Y. Huang Y. Zhang Z. Wang Z. Liu B. Liu C.. (2023). Plant image recognition with deep learning: A review. Comput. Electron. Agric. 212, 108072. doi: 10.1016/j.compag.2023.108072

[B11] Ferentinos K. P. (2018). Deep learning models for plant disease detection and diagnosis. Comput. Electron. Agric. 145, 311–318. doi: 10.1016/j.compag.2018.01.009

[B12] Fuentes A. Yoon S. Kim S. C. Park D. S. (2017). A robust deep-learning-based detector for real-time tomato plant diseases and pests recognition. Sensors 17, 2022. doi: 10.3390/s17092022 28869539 PMC5620500

[B13] Fukui H. Hirakawa T. Yamashita T. Fujiyoshi H. (2019). Attention branch network: Learning of attention mechanism for visual explanation. In VPR 2019: Proceedings of the IEEECVF Conf. Comput. Vis. Pattern Recognit. (CVPR); 2019 Jun 15-20; Long Beach, California, USA, p. 10705–10714. doi: 10.1109/cvpr.2019.01096

[B14] Geetharamani G. Arun Pandian J. (2019). Identification of plant leaf diseases using a nine-layer deep convolutional neural network. Comput. Electr. Eng. 76, 323–338. doi: 10.1016/j.compeleceng.2019.04.011

[B15] Hanes S. P. Collum K. K. Hoshide A. K. Asare E. (2015). Grower perceptions of native pollinators and pollination strategies in the lowbush blueberry industry. Renew. Agric. Food Syst. 30, 124–131. doi: 10.1017/S1742170513000331

[B16] Haralick R. M. Shapiro L. G. (1985). Image segmentation techniques. Comput. Vis. Graph. Image. Process. 29, 100–132. doi: 10.1016/S0734-189X(85)90153-7

[B17] He K. Zhang X. Ren S. Sun J. (2016). Deep residual learning for image recognition. In CVPR 2016: Proceedings of IEEE Conf. Comput. Vis. Pattern Recognit.; 2016 Jun26 -Jul01; Las Vegas, Nevada, USA. p. 770–778. doi: 10.1109/cvpr.2016.90

[B18] Hou Q. Zhou D. Feng J. (2021). Coordinate attention for efficient mobile network design. In CVPR 2021: Proceedings of the IEEE/CVF conference on computer vision and pattern recognition. 2021 Jun 20-25; Nashville, TN, USA; p. 13713–13722. doi: 10.48550/arXiv.2103.02907

[B19] Hu J. Shen L. Sun G. (2018). Squeeze-and-excitation networks. In CVPR 2018: Proceedings of IEEECVF Conf. Comput. Vis. Pattern Recognit.. 2018 Jun 18-22; Salt Lake City, USA; p. 7132–7141. doi: 10.1109/cvpr.2018.00745

[B20] Liang Q. Xiang S. Hu Y. Coppola G. Zhang D. Sun W. (2019). PD2SE-Net: Computer-assisted plant disease diagnosis and severity estimation network. Comput. Electron. Agric. 157, 518–529. doi: 10.1016/j.compag.2019.01.034

[B21] Liu B. Ding Z. Tian L. He D. Li S. Wang H. (2020). Grape leaf disease identification using improved deep convolutional neural networks. Front. Plant Sci. 11. doi: 10.3389/fpls.2020.01082 PMC7373759 32760419

[B22] Liu J. Wang X. (2020a). Early recognition of tomato gray leaf spot disease based on MobileNetv2-YOLOv3 model. Plant Methods 16, 83. doi: 10.1186/s13007-020-00624-2 32523613 PMC7281931

[B23] Liu J. Wang X. (2020b). Tomato diseases and pests detection based on improved yolo V3 convolutional neural network. Front. Plant Sci. 11. doi: 10.3389/fpls.2020.00898 PMC7309963 32612632

[B24] Liu J. Wang X. (2021). Plant diseases and pests detection based on deep learning: a review. Plant Methods 17, 22. doi: 10.1186/s13007-021-00722-9 33627131 PMC7903739

[B25] Liu K. Zhang X. (2022). PiTLiD: identification of plant disease from leaf images based on convolutional neural network. IEEE/ACM Trans. Comput. Biol. Bioinf. 20, 1278–1288. 10.1109/TCBB.2022.3195291 35914052

[B26] Liu W. Wen Y. Yu Z. Yang M. (2016). Large-margin softmax loss for convolutional neural networks. In ICML 2016: Proceedings of international Conference on Machine Learning; 2016 Jun 19-24; New York USA; arXiv:1612.02295. doi: 10.48550/arXiv.1612.02295

[B27] Lu Y. Yi S. Zeng N. Liu Y. Zhang Y. (2017). Identification of rice diseases using deep convolutional neural networks. Neurocomputing 267, 378–384. doi: 10.1016/j.neucom.2017.06.023

[B28] Martinelli F. Scalenghe R. Davino S. Panno S. Scuderi G. Ruisi P.. (2015). Advanced methods of plant disease detection. A review. Agron. Sustain. Dev. 35, 1–25. doi: 10.1007/s13593-014-0246-1

[B29] McGovern K. B. Annis S. L. Yarborough D. E. (2012). Efficacy of organically acceptable materials for control of mummy berry disease on lowbush blueberries in maine. Int. J. Fruit Sci. 12, 188–204. doi: 10.1080/15538362.2011.619350

[B30] Mnih V. Heess N. Graves A. (2014). Recurrent models of visual attention. In NIPS 2014: Proceedings of The Twenty-eighth Annual Conference on Advances in Neural Information Processing Systems 27; 2014 Dec 8-13; Montreal, Quebec, Canada; p.2204-2212. doi: 10.48550/arXiv.1406.6247

[B31] Mohanty S. P. Hughes D. P. Salathé M. (2016). Using deep learning for image-based plant disease detection. Front. Plant Sci. 7. doi: 10.3389/fpls.2016.01419 PMC5032846 27713752

[B32] Nagasubramanian K. Jones S. Singh A. K. Sarkar S. Singh A. Ganapathysubramanian B. (2019). Plant disease identification using explainable 3D deep learning on hyperspectral images. Plant Methods 15, 98. doi: 10.1186/s13007-019-0479-8 31452674 PMC6702735

[B33] nass.usda.gov. Available online at: https://www.nass.usda.gov/ (Accessed 15 November 2023).

[B34] Obsie E. Y. Qu H. Drummond F. (2020). Wild blueberry yield prediction using a combination of computer simulation and machine learning algorithms. Comput. Electron. Agric. 178, 105778. doi: 10.1016/j.compag.2020.105778

[B35] Penman L. N. Annis S. L. (2005). Leaf and flower blight caused by monilinia vaccinii-corymbosi on lowbush blueberry: effects on yield and relationship to bud phenology. Phytopathology® 95, 1174–1182. doi: 10.1094/PHYTO-95-1174 18943470

[B36] Perez L. Wang J. (2017). The effectiveness of data augmentation in image classification using deep learning. arXiv. doi: 10.48550/arxiv.1712.04621

[B37] Picon A. Seitz M. Alvarez-Gila A. Mohnke P. Ortiz-Barredo A. Echazarra J. (2019). Crop conditional Convolutional Neural Networks for massive multi-crop plant disease classification over cell phone acquired images taken on real field conditions. Comput. Electron. Agric. 167, 105093. doi: 10.1016/j.compag.2019.105093

[B38] Qu H. Drummond F. (2018). Simulation-based modeling of wild blueberry pollination. Comput. Electron. Agric. 144, 94–101. doi: 10.1016/j.compag.2017.11.003

[B39] Qu H. Liu G. (2020). Threshold optimized strategy based on improved flower pollination algorithm for unbalanced data. 2020. IEEE 10th. Int. Conf. Intell. Syst. 00, 551–556. doi: 10.1109/is48319.2020.9199930

[B40] Ramesh S. Hebbar R. Niveditha. M. Pooja. R. Prasad Bhat. N. Shashank N.. (2018). Plant disease detection using machine learning. In ICDI3C 2018: Proceedings of Int. Conf. Des. Innov. 3Cs Comput. Commun. Control; 2018 Apr 25-28; Bangalore, India p. 41–45. doi: 10.1109/ICDI3C45473.2018

[B41] Ruder S. (2017). An Overview of Multi-Task Learning in Deep Neural Networks. arXiv. doi: 10.48550/arxiv.1706.05098

[B42] Rumpf T. Mahlein A.-K. Steiner U. Oerke E.-C. Dehne H.-W. Plümer L. (2010). Early detection and classification of plant diseases with Support Vector Machines based on hyperspectral reflectance. Comput. Electron. Agric. 74, 91–99. doi: 10.1016/j.compag.2010.06.009

[B43] Sastry K. S. Zitter T. A. (2014). Plant virus and viroid diseases in the tropics. Epidemiol. Management. 2, 149–480. doi: 10.1007/978-94-007-7820-7_2

[B44] Shorten C. Khoshgoftaar T. M. (2019). A survey on image data augmentation for deep learning. J. big. Data 6, 1–48. doi: 10.1186/s40537-019-0197-0 PMC8287113 34306963

[B45] Su Z. Fang L. Kang W. Hu D. Pietikäinen M. Liu L. (2020) Computer vision-ECCV 2020 in ECCV 2020: Proceedings of 16th European Conference on Computer vision, Glasgow, UK, August 23–28, 2020. 138–155, Proceedings, Part VI. doi: 10.1007/978-3-030-58539-6_9

[B46] Tan M. Le Q. (2019). Efficientnet: Rethinking model scaling for convolutional neural networks. In International conference on machine learning. 2019 Jun 9-15; Long Beach USA, 6105–6114. doi: 10.48550/arxiv.1905.11946

[B47] Taylor L. Nitschke G. (2018). Improving deep learning with generic data augmentation. In 2018 IEEE symposium series on computational intelligence (SSCI). 1542–1547 (IEEE).

[B48] Thangaraj R. Anandamurugan S. Kaliappan V. K. (2021). Automated tomato leaf disease classification using transfer learning-based deep convolution neural network. J. Plant Dis. Prot. 128, 73–86. doi: 10.1007/s41348-020-00403-0

[B49] Vishnoi V. K. Kumar K. Kumar B. (2021). Plant disease detection using computational intelligence and image processing. J. Plant Dis. Prot. 128, 19–53. doi: 10.1007/s41348-020-00368-0

[B50] Waghmare H. Kokare R. Dandawate Y. (2016). Detection and classification of diseases of grape plant using opposite color local binary pattern feature and machine learning for automated decision support system. In SPIN 2016: Proceedings of the 3rd International Conference on Signal Processing and Integrated Networks. 2016 Feb 11-12; Noida, India. 513–518. doi: 10.1109/spin.2016.7566749

[B51] Woo S. Park J. Lee J. Y. Kweon I. S. (2018). Cbam: Convolutional block attention module. In. In ECCV 2018: Proceedings of the 15th European conference on computer vision. 2018 Sep 8-14; Munich, Germany; p. 3–19. doi: 10.1007/978-3-030-01234-2_1

[B52] Xie X. Ma Y. Liu B. He J. Li S. Wang H. (2020). A deep-learning-based real-time detector for grape leaf diseases using improved convolutional neural networks. Front. Plant Sci. 11. doi: 10.3389/fpls.2020.00751 PMC7285655 32582266

[B53] Xu K. Ba J. Kiros R. Cho K. Courville A. Salakhudinov R.. (2015). Show, attend and tell: Neural image caption generation with visual attention. In ICML 2015: Proceedings of the 32nd International Conference on Machine Learning. 2015 Jul 6-11; Lille, France; p. 2048–2057. doi: 10.48550/arxiv.1502.03044

[B54] Zhang Y. Yang Q. (2017). A survey on multi-task learning. IEEE Trans. Knowl. Data Eng. 34, 5586-5609. doi: 10.1109/TKDE.2021.3070203

[B55] Zhang M. Yang Y. Shen F. Zhang H. Wang Y. (2017). Multi-view feature selection and classification for Alzheimer’s Disease diagnosis. Multimedia. Tools Appl. 76, 10761–10775. doi: 10.1007/s11042-015-3173-5

[B56] Zhou J. Zhang Q. Zhang B. (2021). An automatic multi-view disease detection system *via* Collective Deep Region-based Feature Representation. Futur. Gener. Comput. Syst. 115, 59–75. doi: 10.1016/j.future.2020.08.038

