# Supplementary material for: A deep multi-task learning approach to identifying mummy berry infection sites, the disease stage, and severity
Source: Front Plant Sci. 2024 Mar 28;15:1340884. doi: 10.3389/fpls.2024.1340884 (PMC11007028; doi:10.3389/fpls.2024.1340884)
Supplement: Supplementary file 1 [file DataSheet_1.docx]

## Supplementary Tables

**Appendix A**. Structure detail of the four instances of the Deep MTL model.

**Table A1**. Configuration of the Parameter-sharing module for MummyBerryNet-B0.

| Phase  $i$ | Operator  $F_{i}$ | Resolution  $H_{i}\times W_{i}$ | Channels  $C_{i}$ | Layers  $L_{i}$ |
| --- | --- | --- | --- | --- |
| 1 | $Conv3\times3$ | $224 \times224$ | 32 | 1 |
| 2 | $MBConv1, K3\times3$ | $112 \times112$ | 16 | 1 |
| 3 | $MBConv6,K3\times3$ | $112 \times112$ | 24 | 2 |
| 4 | $MBConv6, K5\times5$ | $56 \times56$ | 40 | 2 |
| 5 | $MBConv6, K3\times3$ | $28 \times28$ | 80 | 3 |
| 6 | $MBConv6, K5\times5$ | $14 \times14$ | 112 | 4 |
| 7 | $MBConv6, K5\times5$ | $14 \times14$ | 192 | 5 |
| 8 | $MBConv6, K3\times3$ | $7 \times7$ | 320 | 1 |

**Table A2**. Configuration of the Parameter-sharing module for MummyBerryNet-B1.

| Phase  $i$ | Operator  $F_{i}$ | Resolution  $H_{i}\times W_{i}$ | Channels  $C_{i}$ | Layers  $L_{i}$ |
| --- | --- | --- | --- | --- |
| 1 | $Conv3\times3$ | $240 \times240$ | 32 | 1 |
| 2 | $MBConv1, K3\times3$ | $120 \times120$ | 16 | 2 |
| 3 | $MBConv6,K3\times3$ | $120 \times120$ | 24 | 3 |
| 4 | $MBConv6, K5\times5$ | $60 \times60$ | 40 | 3 |
| 5 | $MBConv6, K3\times3$ | $30 \times30$ | 80 | 4 |
| 6 | $MBConv6, K5\times5$ | $15 \times15$ | 112 | 4 |
| 7 | $MBConv6, K5\times5$ | $8 \times$ 8 | 192 | 5 |
| 8 | $MBConv6, K3\times3$ | $8 \times8$ | 320 | 2 |

**Table A3**. Configuration of the Parameter-sharing module for MummyBerryNet-B2.

| Phase  $i$ | Operator  $F_{i}$ | Resolution  $H_{i}\times W_{i}$ | Channels  $C_{i}$ | Layers  $L_{i}$ |
| --- | --- | --- | --- | --- |
| 1 | $Conv3\times3$ | $260 \times60$ | 32 | 1 |
| 2 | $MBConv1, K3\times3$ | $130\times130$ | 16 | 2 |
| 3 | $MBConv6,K3\times3$ | $65 \times65$ | 24 | 3 |
| 4 | $MBConv6, K5\times5$ | $33 \times33$ | 48 | 3 |
| 5 | $MBConv6, K3\times3$ | $17 \times17$ | 88 | 4 |
| 6 | $MBConv6, K5\times5$ | $17 \times17$ | 120 | 4 |
| 7 | $MBConv6, K5\times5$ | $9 \times9$ | 208 | 5 |
| 8 | $MBConv6, K3\times3$ | $9 \times9$ | 352 | 2 |

**Table A4**. Configuration of the Parameter-sharing module for MummyBerryNet-B3.

| Phase  $i$ | Operator  $F_{i}$ | Resolution  $H_{i}\times W_{i}$ | Channels  $C_{i}$ | Layers  $L_{i}$ |
| --- | --- | --- | --- | --- |
| 1 | $Conv3\times3$ | $300\times300$ | 32 | 1 |
| 2 | $MBConv1, K3\times3$ | $150 \times150$ | 24 | 2 |
| 3 | $MBConv6,K3\times3$ | $75\times75$ | 32 | 3 |
| 4 | $MBConv6, K5\times5$ | $38 \times38$ | 48 | 3 |
| 5 | $MBConv6, K3\times3$ | $19 \times19$ | 96 | 5 |
| 6 | $MBConv6, K5\times5$ | $19 \times19$ | 136 | 5 |
| 7 | $MBConv6, K5\times5$ | $10 \times10$ | 232 | 6 |
| 8 | $MBConv6, K3\times3$ | $10 \times10$ | 384 | 2 |

**Table A5**. The number of parameters and computational cost (FLOP) of the four instances of MummyBerryNet (M stands for million) as well as the five counterparts. The number of parameters were counted for both the Parameter-sharing and Task-specific modules.

| Models | Number of parameters | FLOPs |
| --- | --- | --- |
| MummyBerryNet-B0 | 21.47 M | 0.85 G |
| MummyBerryNet-B1 | 23.98 M | 1.13 G |
| MummyBerryNet-B2 | 25.26 M | 1.46 G |
| MummyBerryNet-B3 | 28.35 M | 1.86 G |
| AlexNet | 61.1 M | 0.72 G |
| VGG16 | 138.36 M | 15.48 G |
| ResNet50 | 25.56 M | 4.11 G |
| MobileNetV2 | 3.5 M | 0.32 G |
| EfficientNet-B0 | 5.3 M | 0.39G |

**Appendix B**. Settings of the five state-of-the-art models and the four instances of MummyBerryNet for performance comparison.

**Table B1**. Performance evaluation experiment. The input images of the network were converted with a fixed size to meet the input size requirements of CNN networks.

| Model | Input size |
| --- | --- |
| MummyBerryNet-B0 | $224\times224$ |
| MummyBerryNet -B1 | $240\times240$ |
| MummyBerryNet -B2 | $260\times260$ |
| MummyBerryNet -B3 | $300\times300$ |
| AlexNet | $224\times224$ |
| VGG16 | $224\times224$ |
| ResNet50 | $224\times224$ |
| MobileNetV2 | $224\times224$ |

**Table B2**. Performance comparisons between the four instances of MummyBerryNet and the five state-of-the-art CNNs. The best model performance is shown in bold. Italics indicate the proposed method and its performance.

| Models | Site | | Stage | | Severity | |
| --- | --- | --- | --- | --- | --- | --- |
|  | Accuracy | F1-score | Accuracy | F1-score | Accuracy | F1-score |
| AlexNet | 82.62 | 82.20 | 83.89 | 83.85 | 83.44 | 83.07 |
| VGG16 | 82.88 | 82.74 | 84.37 | 84.32 | 83.44 | 83.06 |
| ResNet50 | 83.40 | 83.42 | 84.37 | 84.36 | 83.21 | 82.90 |
| MobileNetV2 | 83.66 | 83.41 | 84.60 | 84.56 | 82.27 | 81.35 |
| EfficientNetb0 | 89.83 | 89.23 | 91.90 | 91.90 | 90.62 | 87.63 |
| EfficientNetb1 | 89.55 | 88.82 | 91.65 | 91.57 | 90.88 | 88.36 |
| EfficientNetb2 | 89.55 | 88.89 | 90.87 | 90.89 | 90.88 | 88.71 |
| EfficientNetb3 | 90.98 | 90.09 | 91.39 | 91.45 | 90.62 | 87.47 |
| *MummyBerryNet-B0* | **96.81** | **97.03** | 96.26 | 96.83 | 95.91 | **92.31** |
| *MummyBerryNet-B1* | 96.52 | 96.56 | **97.13** | **97.68** | **96.51** | 92.04 |
| *MummyBerryNet-B2* | 95.63 | 95.50 | 95.68 | 96.25 | 95.91 | 92.14 |
| *MummyBerryNet-B3* | 96.52 | 96.72 | 96.55 | 97.04 | 95.91 | 91.12 |

**Table B3.** The disease detection accuracy of each instance of MummyBerryNet on each task under the STL scheme compared to the same task performed under the MTL scheme (number in the brackets). Transfer learning was applied to both STL and MTL learning framework employed in this ablation experiment.

| Models | Site identification | Stage classification | Severity estimation |
| --- | --- | --- | --- |
| MummyBerryNet-B0 | 93.68 (96.81) | 91.84 (96.26) | 93.16 (95.91) |
| MummyBerryNet-B1 | 92.89 (96.52) | 93.41 (97.13) | 93.41 (96.51) |
| MummyBerryNet-B2 | 93.16 (95.63) | 92.36 (95.68) | 93.41 (95.91) |
| MummyBerryNet-B3 | 92.89 (96.52) | 92.89 (96.55) | 93.11 (95.91) |

**Table B4.** The disease detection accuracy of each instance of MummyBerryNet on each task without transfer learning compared to the same task conducted with transfer learning (number in the brackets). The MTL learning scheme was applied to both two scenarios.

| Models | Site identification | Stage classification | Severity estimation |
| --- | --- | --- | --- |
| MummyBerryNet-B0 | 75.53 (96.64) | 79.59 (94.74) | 77.2 (96.10) |
| MummyBerryNet-B1 | 65.86 (95.82) | 65.38 (96.36) | 72.34 (96.36) |
| MummyBerryNet-B2 | 66.16 (96.10) | 65.38 (95.28) | 72.46 (96.36) |
| MummyBerryNet-B3 | 61.33 (95.82) | 59.47 (95.82) | 54.71 (96.05) |

**Appendix C**. Enhanced visualization of model comparison and exploration.

| Models | Scenario A | Scenario B | Scenario C |
| --- | --- | --- | --- |
| Input image | 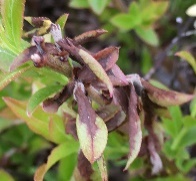 | 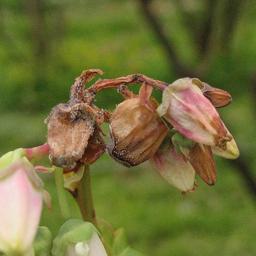 | 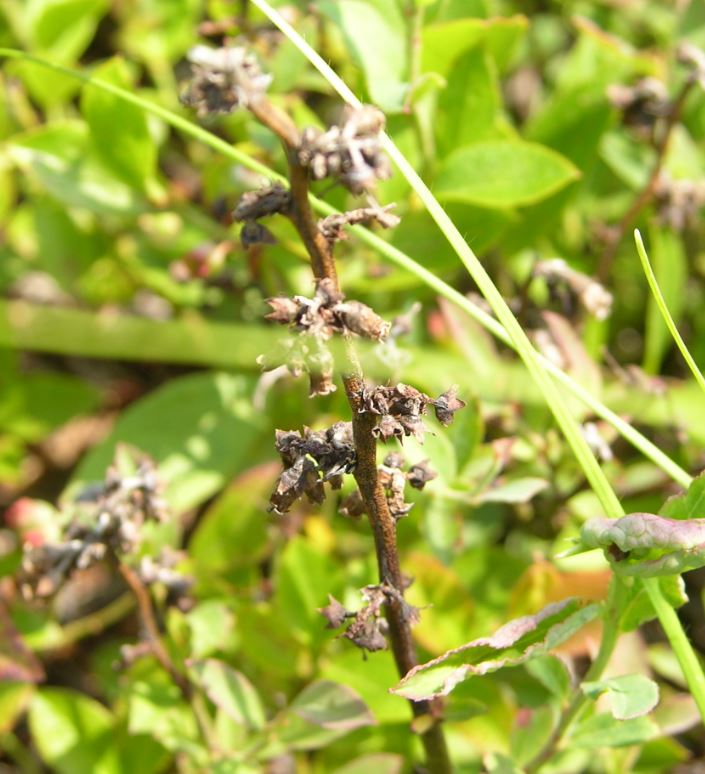 |
| ResNet50 | 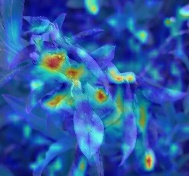 | 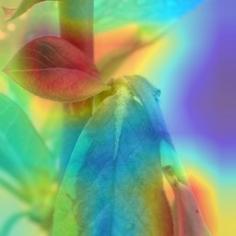 | 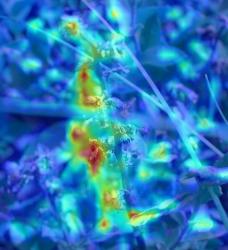 |
| Alexnet | 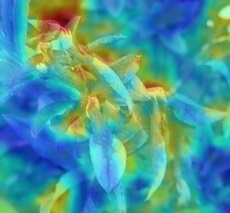 | 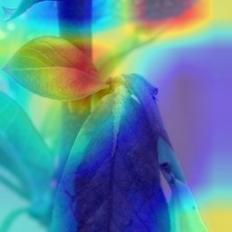 | 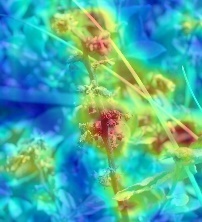 |
| VGG16 | 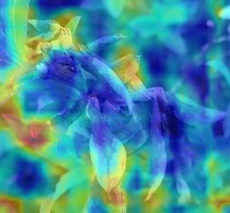 | 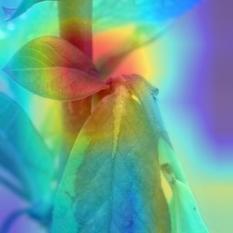 | 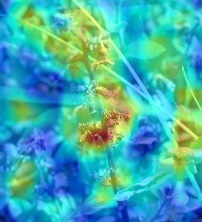 |
| Mobilenetv2 | 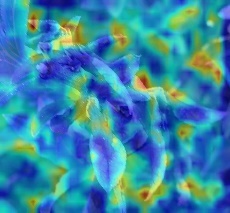 | 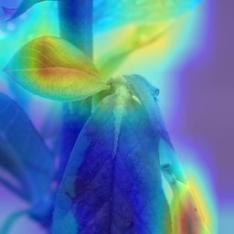 | 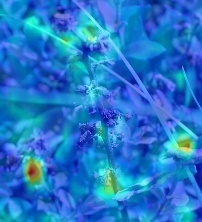 |
| MummyBerryNet | 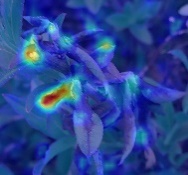 | 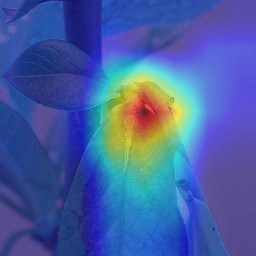 | 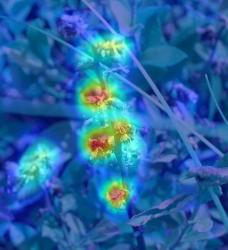 |

**Figure C1.** Visual comparison of disease detection between MummyBerryNet and the four state-of-the-art CNN models, ResNet50, Alexnet, VGG16 and Mobilenetv2. Three different scenarios (A, B and C) were chosen, representing complex (A), simple (B) background and occlusion (C).


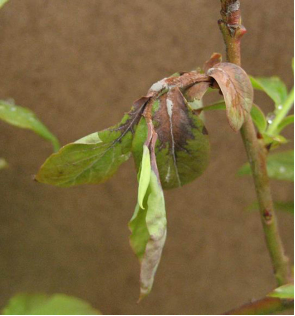

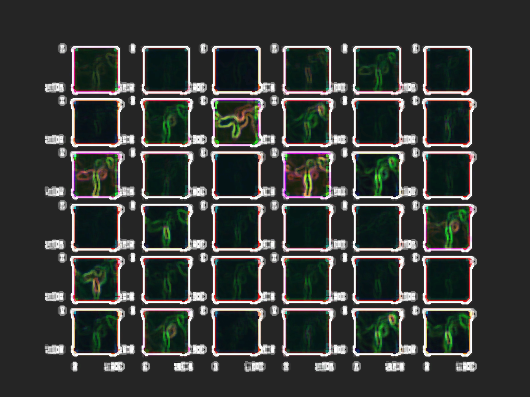

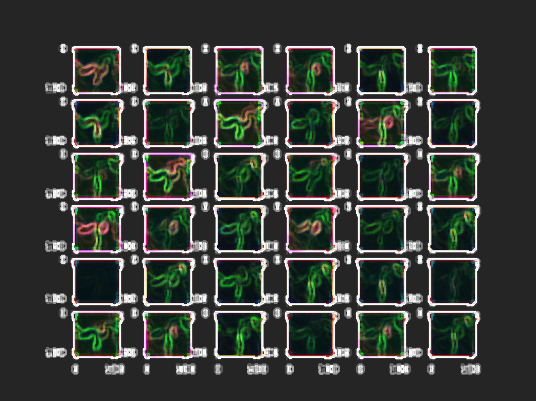


**Figure C2**. Visualized feature extraction comparison between scenarios where multiple attention mechanisms were applied (right) and no attention mechanism was used (middle). Left is the original input image.
